# The bacterial succession and its role in flavor compounds formation during the fermentation of cigar tobacco leaves

**DOI:** 10.1186/s40643-023-00694-9

**Published:** 2023-10-31

**Authors:** Hongyang Si, Kun Zhou, Tingyi Zhao, Bing Cui, Fang Liu, Mingqin Zhao

**Affiliations:** College of Tobacco Science, Flavors and Fragrance Engineering & Technology Research Center of Henan Province, Henan Agricultural University, No.218 Ping An Avenue, Zhengdong New District, Zhengzhou, 450046 Henan China

**Keywords:** Cigar tobacco leaves, Fermentation, Bacterial community, Flavor substances, Co-occurrence network

## Abstract

Fermentation is the key process required for developing the characteristic properties of cigar tobacco leaves, complex microorganisms are involved in this process. However, the microbial fermentation mechanisms during the fermentation process have not been well-characterized. This study investigated the dynamic changes in conventional chemical composition, flavor compounds, and bacterial community during the fermentation of cigar tobacco leaves from Hainan and Sichuan provinces in China, as well as the potential roles of bacteria. Fermentation resulted in a reduction of conventional chemical components in tobacco leaves, with the exception of a noteworthy increase in insoluble protein content. Furthermore, the levels of 10 organic acids and 19 amino acids showed a significant decrease, whereas the concentration of 30 aromatic substances exhibited a unimodal trend. Before fermentation, the bacterial community structures and dominant bacteria in Hainan and Sichuan tobacco leaves differed significantly. As fermentation progressed, the community structures in the two regions became relatively similar, with *Delftia*, *Ochrobactrum*, *Rhodococcus*, and *Stenotrophomonas* being dominant. Furthermore, a total of 12 functional bacterial genera were identified in Hainan and Sichuan tobacco leaves using bidirectional orthogonal partial least squares (O2PLS) analysis. *Delftia*, *Ochrobactrum*, and *Rhodococcus* demonstrated a significant negative correlation with oleic acid and linoleic acid, while *Stenotrophomonas* and *Delftia* showed a significant negative correlation with undesirable amino acids, such as Ala and Glu. In addition, *Bacillus* showed a positive correlation with benzaldehyde, while *Kocuria* displayed a positive correlation with 2-acetylfuran, isophorone, 2, 6-nonadienal, and β-damascenone. The co-occurrence network analysis of microorganisms revealed a prevalence of positive correlations within the bacterial network, with non-abundant bacteria potentially contributing to the stabilization of the bacterial community. These findings can improve the overall tobacco quality and provide a novel perspective on the utilization of microorganisms in the fermentation of cigar tobacco leaves.

**Graphical Abstract:**

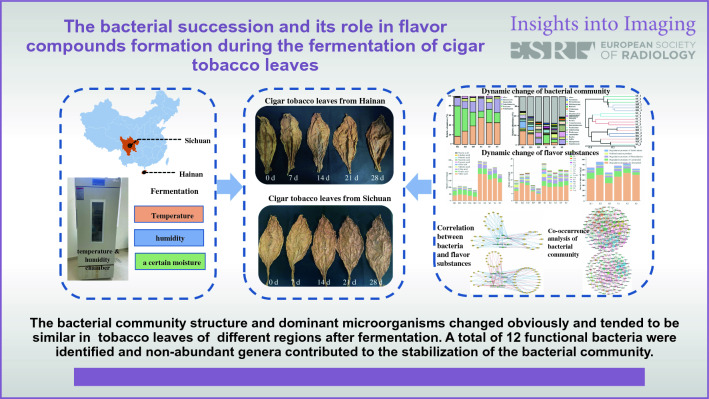

**Supplementary Information:**

The online version contains supplementary material available at 10.1186/s40643-023-00694-9.

## Introduction

The tobacco plant (*Nicotiana tabacum* L.) is one of the most economically important non-food crops cultivated in the world (Ye et al. 2019). China is the largest producer and consumer of tobacco, accounting for approximately one-third of global consumption each year (Liu et al. 2015). Cigars, a type of tobacco product crafted by rolling cigar tobacco leaves, exhibit the characteristics of rich aroma and high smoke concentration. The cigar tobacco planting area in China has grown significantly from 280 to 2667 hectares during 2019–2023, and the proportion of domestically cigar raw materials has increased from less than 6% to over 50% in 2023 (Yue et al. 2023). The main steps of cigar manufacturing include cultivation, air-curing, fermentation, and rolling (Zhao et al. 2022b). Fermentation is a crucial step in improving the quality of cigar tobacco leaves (Yang et al. 2018). During the fermentation process, microbial activity, enzyme catalysis, and complex chemical reactions lead to the decomposition of proteins, starch, and other substances, producing a range of flavor compounds, such as acids, ketones, aldehydes, and alcohols (Dai et al. 2020; Zheng et al. 2022a, b). Organic acids and amino acids serve as flavor components or precursors in tobacco leaves, while aromatic substances contribute to the overall quality of tobacco and directly influence sensory characteristics (Hu et al. 2022b). Recent research has revealed that elevated levels of trimethylamine in tobacco leaves are responsible for the irritating ammonia flavor in cigars (Shi et al. 2023). Fan et al. (2023) identified and characterized 613 volatile metabolites in cigar tobacco leaves during the fermentation process, highlighting benzaldehyde as the primary differentiating metabolite, known for its distinct almond and cherry aromas. Similar to fermented foods, previous reports have suggested the microbial contribution to the formation of flavor compounds (Loew 1899). Nonetheless, in comparison with extensively investigated fermented foods, the microbial fermentation mechanisms specific to cigar tobacco leaf fermentation remain less explored.

Previous studies using culture-dependent methods have elucidated the capacity of microorganisms isolated from tobacco leaves to degrade nicotine, tobacco-specific nitrosamines, and other undesirable components while also exhibiting the ability to convert lutein and chlorogenic acid into aromatic substances (Ma et al. 2016; Maldonado-Robledo et al. 2003; Wei et al. 2014; Zhong et al. 2010). However, only approximately 1% of the microbiota from high-diversity environments can be cultivated using culture-dependent techniques (Giraffa and Neviani 2001). With the development of new generation of high-throughput sequencing technology (Shendure and Ji 2008), recent studies have increasingly focused on microbial community composition and diversity in tobacco leaves (Zheng et al. 2022a; Xing et al. 2021), but limited information is available about the dynamic changes and specific role of these microorganisms in the fermentation process. Thus, it is meaningful to explore the succession and function of the microorganisms responsible for the formation of characteristic flavor compounds to achieve a comprehensive understanding of the fermentation process and ensure the consistent quality of fermented leaves. Bidirectional orthogonal partial least squares (O2PLS) modeling is an effective approach that has been extensively implemented in food fermentation to elucidate the relationship between microorganisms and flavor substances (El Bouhaddani et al. 2016). For instance, Guan et al. (2022) investigated microbial succession and flavor changes during suansun fermentation and found that *Lactobacillus*, *Clostridium*_*sensu_stricto_1*, *Enterobacter*, and *Leuconostoc* played crucial roles in the unique flavor formation of suansun using O2PLS. In addition, Li et al. (2019) explored microbiota dynamics and their correlations with the major chemical compounds during the manufacture of Fu brick tea, and 19 bacterial genera were identified as core functional genera linked to the metabolism of chemical compounds using O2PLS. Moreover, Zang et al. (2020) investigated changes in free amino acid, organic acid, and free fatty acid profiles in Chinese traditional fermented fish and observed that 2 bacteria and 11 fungal genera were responsible for the formation of flavor compounds. To the best of our knowledge, there is no research on the application of O2PLS analysis in investigating the relationship between microorganisms and flavor compounds during tobacco fermentation.

Previous research has demonstrated extensive regional variation in both microbial communities and flavor profiles of cigar tobacco leaves (Zheng et al. 2022a). However, the effect of fermentation on microbial community and flavor substances in cigar tobacco leaves from diverse regions has yet to be fully elucidated. Therefore, in this study, we aimed to address this gap by selecting tobacco leaves from two distinct regions in China, namely, Hainan and Sichuan, and characterizing the assembly and dynamics of the bacterial community, as well as interactions among microbial communities during the fermentation process. Simultaneously, we investigated changes in conventional chemical components and flavor substances of the leaves. Based on these results, the specific relationship between microbiota and flavor components during the fermentation process was investigated, and functional bacteria were revealed using O2PLS. The findings of this study will greatly advance our understanding of the fermentation mechanisms involved in cigar tobacco leaf fermentation, and provide a crucial theoretical foundation for the production of high-quality cigar tobacco.

## Materials and methods

### Experimental design and sample collection

A variety of Haiyan 103 grown in Wuzhishan (18°46′N, 109°31′E), Hainan province (H), and a variety of Chuanxue No.1 grown in Dazhou (32°20′N, 108°31′E), Sichuan province (S), were selected as experimental materials for this study. In Wuzhishan, the field was red sandy loam soil with 16.2 g/kg organic matter, 63.16 mg/kg of available nitrogen, 26.27 mg/kg of available phosphorus, 124.61 mg/kg of rapidly available potassium, and a pH of 5.9 (Liu et al. 2021a). The Dazhou field had a sandy loam soil texture with 20.28 g/kg organic matter, 92 mg/kg of available nitrogen, 20.32 mg/kg of available phosphorus, 98.99 mg/kg of rapidly available potassium, and a pH of 6.13 (Jin et al. 2023). The fermentation process of tobacco leaves was performed as described by Liu et al. (2021b), with a little modification. After harvesting, the tobacco leaves were subjected to the curing process in an air-drying shelter. Subsequently, 15 kg of middle leaves were randomly selected from the shelter and promptly transported to the laboratory of Henan Agricultural University for fermentation. The moisture content of tobacco leaves was adjusted to 25% using sterile water before fermentation. Next, the leaves were placed at 30 °C and 80% relative humidity for 48 h to reach water equilibrium. Following this, 5 kg of tobacco leaves were stacked evenly in separate cabinets with a temperature of 42 °C and relative humidity of 90%. Each sample contained three independent biological replicates. The overall fermentation period lasted for 28 days, during which samples were collected every 7 days. A total of 30 samples were collected from five independent fermentation stages at 0 (H1 and S1), 7 (H2 and S2), 14 (H3 and S3), 21 (H4 and S4), and 28 (H5 and S5) days. The samples were packaged in sterile bags and stored at 80 °C until further analysis.

### Determination of chemical compositions in tobacco samples

The analysis of total sugar and reducing sugar, total nitrogen, nicotine, and water-insoluble protein was performed using the continuous flow methods YC/T159-2002, YC/T161-2002, YC/T160-2002, and YC/249-2008, respectively. Water-soluble protein content was determined using Coomassie Brilliant Blue staining. The free amino acid content was analyzed using the amino acid analyzer method YC/T282-2009. The non-volatile organic acid was determined using the gas chromatography method YC/T 288-2009.

### Determination of aromatic components in tobacco samples

The simultaneous distillation extraction technique was adopted to extract aromatic substances from tobacco leaves, and gas chromatography–mass spectrometry (GC–MS) was utilized to determine the content of aromatic substances in cigar tobacco leaves (Yun et al. 2013). In detail, 20 g of cigar tobacco powder was mixed with 2 g of citric acid and 1000 ml of deionized water in a 2000 ml flat-bottomed flask. Subsequently, 40 ml dichloromethane and 1 ml internal standard (phenethyl acetate) were added to a 250 ml flask, which was heated in a 60 °C water bath for simultaneous distillation and extraction for 2.5 h. Afterwards, the dichloromethane phase was collected and concentrated to 1 ml using a rotary evaporator for GC–MS analysis. The GC–MS system used in this study was Turbo Mass, equipped with DB-5 column (30 m × 0.25 mm × 0.25 μm). The gas chromatographic conditions were as follows: the initial temperature was set at 40 °C for 2 min, followed by a temperature ramping rate of 4 °C/min until reaching 250 °C, which was then maintained for 10 min. The interface temperature was maintained at 290 °C.

### DNA extraction and sequencing

Microorganisms in the tobacco leaves were collected according to the method described by Lyu et al. (2013). DNA was extracted from 0.5 g samples with the E.Z.N.A. Soil DNA Kit (Omega Bio-tek, Norcross, GA, USA) following the manufacturer’s instructions. The quality and quantity of the extracted DNA were estimated using a NanoDrop 2000 spectrophotometer (Thermo Scientific, Waltham, MA, USA), and the extraction quality was assessed through 1% agarose gel for further amplification generation. The primers 799F (5’-AACMGGATTAGATACCCKG-3’) and 1193R (5’-ACGTCATCCCCACCTTCC-3’) were used as previously described in Wang et al. (2018) to amplify the V5–V7 region of the bacterial 16S rRNA gene. The polymerase chain reaction (PCR) products were then extracted using a 2% agarose gel and purified using the AxyPrep DNA Gel Extraction Kit (Axygen Inc., Union City, CA, USA). Subsequently, sequencing of the purified DNA samples was performed on the Illumina MiSeq PE300 platform at Shanghai Majorbio Bio-pharm Technology Co., Ltd. (Shanghai, China).

### Analysis of microorganisms in cigar tobacco leaves

After removing raw reads with low quality and short length, operational taxonomic units (OTUs) were clustered with ≥ 97% similarity using UPARSE (version 7.0, http://drive5.com/uparse/) (Edgar 2013). OTU taxonomic information on bacteria was obtained using the Ribosomal Database Project (RDP) Classifier with a confidence threshold of 70% against the 16S rRNA database (Schloss et al. 2009). After taxonomic assignment, random sampling was employed to equalize the sequence counts across all samples. The original OTU counts were subsequently normalized to an OTU table of relative abundance values before statistical analysis. The dominant bacteria with relative abundances exceeding 1% at phylum and genus levels were analyzed. Venn diagrams were generated to explore the distribution patterns of bacteria at the genus level. Non-metric multidimensional scaling (NMDS) based on Bray–curtis distance algorithm and analysis of similarity test (ANOSIM) was used to determine similarities in phyllosphere bacterial communities. Cluster analysis was calculated using Unweighted Pair-group Method with Arithmetic Mean (UPGMA) based on average Bray–curtis distance. The microbial taxa in each fermentation stage were analyzed using the linear discriminant analysis effect size (LEfSe) algorithm with a threshold LDA score of 4.0. The analysis of 16S sequencing data was conducted on the Majorbio cloud platform (Ren et al. 2022).

### Statistical analysis

Principal component analysis (PCA) and Hierarchical cluster analysis (HCA) were conducted to analyze the dynamic changes in conventional chemical composition factors levels using SIMCA 14.1 software (Wu et al. 2019). The changes in flavor substances were visualized through a heatmap and generated using TBtools (v2.001) (Chen et al. 2020a), and data were clustered in terms of Euclidian distance and each row was scaled to normalize. To identify core functional bacteria, correlations between the top 50 bacteria and flavor compounds were evaluated using O2PLS analysis (Trygg 2010). O2PLS modeling was conducted using SIMCA 14.1 software, and the interaction networks were visualized using Cytoscape (version 3.5.1). The co-occurrence patterns within the tobacco bacterial community were analyzed using the Integrated network analysis pipeline (iNAP) (Feng et al. 2022). OTUs with relative abundances greater than 0.1% were used for the network construction-based pair Pearson correlation coefficients, with a threshold of 0.95 based on the random matrix theory approach. Network topological properties, i.e., within-module connectivity (Zi) and among-module connectivity (Pi), which represent the ecological attributes of the network nodes were also calculated using iNAP (Deng et al. 2012). Finally, the co-occurrence network was visualized using Gephi (version 0.9.7). IBM SPSS statistics 26.0 was used for one-way analysis of variance (ANOVA), and *p* value calculation; significant differences were evaluated using LSD multiple comparison test. All results are expressed as mean ± standard error of the mean, and statistical significance was set at *p* < 0.05.

## Results and discussion

### Dynamic changes of conventional chemical composition during the fermentation process of cigar tobacco leaves

During the fermentation process, dynamic changes occurred in the conventional chemical compounds, including total sugar, reducing sugar, total nitrogen, nicotine, water-soluble protein, and water-insoluble protein (Table 1). These compounds are commonly used as indicators of tobacco leaf quality. Total sugar is a critical factor that determines the smoke sweetness (Chen et al. 2021) while reducing sugar positively affects flavor and aroma (Marija et al. 2020). Notably, the total sugar and reducing sugar contents of Hainan tobacco leaves were significantly higher than those of Sichuan tobacco leaves. Throughout the fermentation, an increase in total sugar content was observed during the 7–14 days and 0–7 days of fermentation in Hainan and Sichuan, respectively, likely due to the degradation of starch and subsequent rise in water-soluble sugar content (Zong et al. 2020). After 28 days of fermentation, there was a significant decrease in the total sugar and reducing sugar contents (*p* < 0.05). Particularly in Sichuan tobacco leaves, which exhibited a reduction rate of 62.69% and 84.85% in total sugar and reducing sugar, respectively, with the reducing sugar content being almost completely depleted. In general, cigar tobacco leaves display lower sugar content and higher nitrogen compound levels compared to flue-cured tobacco. The disappearance of sugars in the leaves can predominantly attributed to Maillard reactions between sugars and amino compounds, along with caramelization reactions involving oxidation, dehydration, isomerization, and polymerization (Gunina and Kuzyakov 2015). These reactions contribute to the formation of aromatic substances.

**Table 1:** Dynamic changes of conventional chemical compounds during fermentation process

Compounds	Hainan					Sichuan				
	H1 (0 d)	H2 (7 d)	H3 (14 d)	H4 (21 d)	H5 (28 d)	S1 (0 d)	S2 (7 d)	S3 (14 d)	S4 (21 d)	S5 (28 d)
Total sugar (%)	1.11 ± 0.12a	0.88 ± 0.09b	0.94 ± 0.15ab	0.94 ± 0.02ab	0.92 ± 0.03b	0.67 ± 0.03a	0.72 ± 0.08a	0.42 ± 0.07b	0.38 ± 0.07b	0.25 ± 0.06c
Reducing sugar (%)	0.76 ± 0.06a	0.55 ± 0.03c	0.59 ± 0.03bc	0.64 ± 0.02b	0.58 ± 0.05bc	0.33 ± 0.02a	0.31 ± 0.01a	0.08 ± 0.01b	0.09 ± 0.02b	0.05 ± 0.01c
Nicotine (%)	4.24 ± 0.12a	4.46 ± 0.09a	4.27 ± 0.07a	3.34 ± 0.33b	2.74 ± 0.21c	2.64 ± 0.3a	2.06 ± 0.07b	1.69 ± 0.07c	1.82 ± 0.07bc	1.70 ± 0.1c
Total nitrogen (%)	3.02 ± 0.21b	3.11 ± 0.22ab	3.33 ± 0.11a	2.69 ± 0.1c	2.70 ± 0.03c	3.85 ± 0.05a	3.60 ± 0.19a	3.82 ± 0.02a	3.81 ± 0.02a	3.63 ± 0.24a
Water-soluble protein (%)	4.68 ± 0.16ab	4.48 ± 0.25bc	5.02 ± 0.11a	4.51 ± 0.3bc	4.16 ± 0.15c	4.37 ± 0.15a	3.88 ± 0.24b	3.50 ± 0.23c	3.19 ± 0.09c	2.76 ± 0.26d
Water-insoluble protein (mg/g)	6.76 ± 0.28bc	6.52 ± 0.30c	7.07 ± 0.06b	7.15 ± 0.20b	7.75 ± 0.25a	7.27 ± 0.41d	7.75 ± 0.27c	9.07 ± 0.20a	8.22 ± 0.16b	8.54 ± 0.07b

Values are mean ± standard error of the mean; (*n* = 3). Statistical significance was assessed by one-way ANOVA followed by LSD multiple comparison test. Different lowercase letters indicate significant difference (*p* < 0.05)

The content of nicotine and total nitrogen in tobacco leaves is associated with physical characteristics and smoke concentration. As fermentation preceded, the nicotine content gradually decreased from 4.24% ± 0.12% in H1 to 2.74% ± 0.21% in H5 and from 2.64 ± 0.30 in S1 to 1.70% ± 0.10% in S5 (*p* < 0.05), representing a reduction of 35.38% and 35.61%, respectively. The decline in nicotine can be attributed to a stepwise dehydrogenation and oxidation process. During the fermentation, a portion of nicotine undergoes conversion into nicotine acid, while another portion transforms into compounds similar to nicotine (Frankenburg 1948). Moreover, the degradation of microorganisms is another contributing factor to the reduced nicotine levels. Previous studies have reported the nicotine degradation ability of microorganisms isolated from tobacco, including *Pseudomonas* sp., *Rhodococcus* sp., *Ochrobactrum* sp., and *Sphingomona*s sp. (Gong et al. 2016; Mu et al. 2020; Yuan et al. 2005). The total nitrogen content in Hainan tobacco leaves showed a unimodal trend and it significantly decreased by 10.60% after fermentation, while there was a fluctuation change of total nitrogen content in Sichuan tobacco leaves which decreased by 4.93% after fermentation. The overall changes in nitrogen compounds are due to the loss of ammonia, amino acids, nicotine, and unidentified nitrogen compounds (Mckee 2010).

The water-soluble protein significantly decreased from H1 (4.68 ± 0.16 mg/g) to H5 (4.16 ± 0.15 mg/g) (*p* < 0.05), and from S1 (4.37 ± 0.15 mg/g) to S5 (2.76 ± 0.26 mg/g) (*p* < 0.05). The decline in water-soluble protein could be associated with the hydrolysis exerted by proteases that remain in the leaves after curing or are secreted by microorganisms, yielding high amounts of free amino acids (Dai et al. 2020). In contrast, the water-insoluble protein content in Hainan tobacco leaves decreased slightly from day 0 to day 7, and then demonstrated a tendency to increase by the end (*p* < 0.05). Moreover, the water-insoluble protein content in Sichuan tobacco leaves significantly increased from 7.27% ± 0.41% in S1 to 8.54% ± 0.07% in S5 (*p* < 0.05). The observed increase in protein content could be attributed to the inherent stable polymer structures of water-insoluble proteins, which remain relatively unchanged during the fermentation process (Gaines and Miles 1975); conversely, the total dry matter experienced a reduction, with a corresponding increase in the proportion of protein. Moreover, the increase in insoluble protein indicates a successful fermentation, while samples that failed to ferment exhibit small or no changes in insoluble protein content (Frankenburg 1950).

**Fig. 1:**
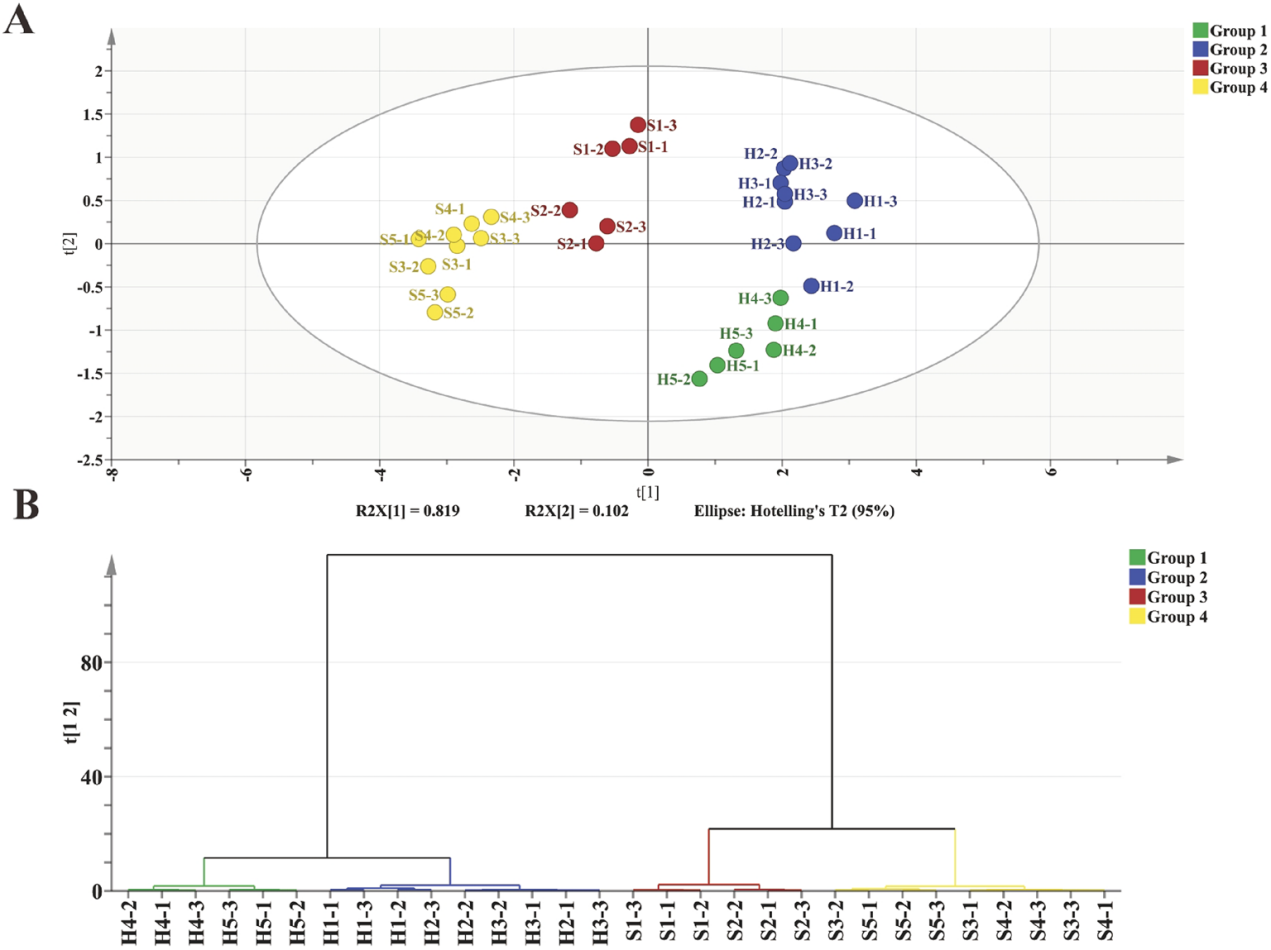
Comparison of the dynamic conventional chemical compounds during the fermentation. **A** Score scatter plot for PCA model; **B** HCA plots based on PCA modeling

PCA and HCA analyses were conducted to reveal the overall variations in conventional chemical components resulting from fermentation. In the PCA score scatterplot (Fig. 1A), the first two principal components explained 81.9% and 10.2% of the variation, respectively. Results of PCA demonstrated differences between Hainan and Sichuan samples throughout the fermentation process. The HCA analysis classified the samples into four groups: H1–H2–H3, H4–H5, S1–S2, and S3–S4–S5 (Fig. 1B). This classification suggested that there were notable changes in the Hainan samples between 14th and 21st day, while a great change was observed in the Sichuan samples between 7th and 14th days. These observations suggest that significant changes in the chemical composition primarily occurred within 7th and 21st days, which is considered as a critical phase for the development of tobacco quality. A previous study suggested that fermentation parameters are thought to distinctly reflect microbial succession in the fermentation (Tan et al. 2019). In our study, the distinct and dynamic profiles observed in the Hainan and Sichuan fermentation process appear to be the result of the intrinsic characteristics of each sample, as well as the contribution of different microbial succession rates.

### Dynamic changes of flavor components during the fermentation process of cigar tobacco leaves

In addition to conventional chemical components, we also analyzed 59 flavor substances content in tobacco leaves during the fermentation process, including 10 non-volatile organic acids, 19 free amino acids, and 30 volatile aromatic components.

**Fig. 2:**
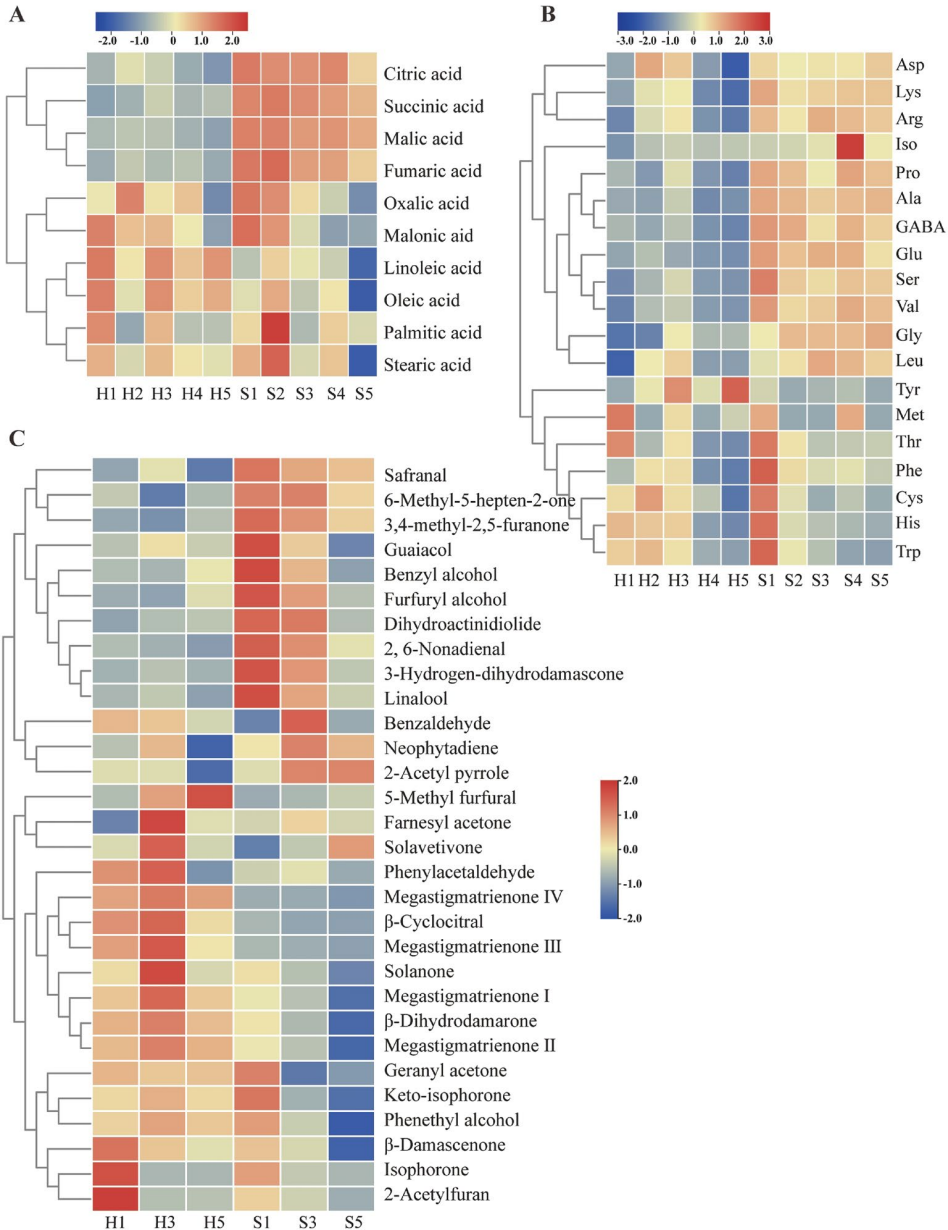
Heatmap analysis of 10 organic acids (**A**), 19 amino acids (**B**), and 30 aromatic compounds (**C**) during the fermentation. Legends show the log-transformed scores of their content. A color-coded scale grading from blue to red corresponds to the content of flavor substances from low to high

Non-volatile organic acids play a critical role in pH regulation and the development of fragrance in flue gas, subsequently influencing the taste and smoke aroma (Xiang et al. 2010). Ten non-volatile organic acids, namely, malic acid, oxalic acid, citric acid, malonic acid, succinic acid, fumaric acid, oleic acid, linoleic acid, palmitic acid, and stearic acid, were detected in this work (Fig. 2A, Additional file 1: Table S1). Results showed that the content of total organic acids in Sichuan tobacco leaves was significantly higher than in Hainan leaves throughout the fermentation process (*p* < 0.05), possibly due to regional altitude variations (Yang et al. 2005). The initial total concentration of organic acids in Hainan was 97.67 ± 0.97 mg/g, then it peaked at 112.95 ± 2.28 mg/g on day 7 and subsequently decreased to 77.94 ± 2.3 mg/g by the end of fermentation. The total organic acids content in S1 was 298.02 ± 3.86 mg/g and significantly decreased to 196.57 ± 1.69 mg/g in S5 (*p* < 0.05). Malic acid was the predominant organic acid in tobacco leaves, accounting for 41.23% and 68.33% of the total organic acids content in H1 and S1, respectively, followed by oxalic acid and citric acid. Compared with H1, H5 showed 23.64%, 14.46%, and 26.23% decreases in malic, oxalic, and citric acids, respectively. In S5, malic acid, oxalic acid, and citric acid declined by 32.80%, 26.74%, and 49.05%, respectively. During the fermentation, most of the absent organic acids were typically converted into carbon dioxide and water. The oxidation of higher molecular-weight organic acids could also generate lower molecular-weight acids, although to a lesser extent. The decrease in malic acid and citric acid observed after fermentation is consistent with previous studies (Di Giacomo et al. 2007). However, the variation trend of oxalic acid was diverse in different research (Di Giacomo et al. 2007; Hu et al. 2022b). In general, oxalic acid is considered as an undesirable end-product of oxidative metabolic reactions in leaves (Vickery 1950). Hence, the reduction of oxalic acid content in our study indicates an improvement in leaf quality following fermentation. The concentrations of the remaining seven organic acids were below 10 mg/g, with linoleic acid and stearic acid particularly below 1 mg/g. Despite their relatively low abundance, these acids exert noticeable effects on the quality of tobacco leaves. Notably, oleic acid and linoleic acid, both unsaturated fatty acids, contribute to increased irritation and astringency in tobacco leaves (Hu et al. 2023). Consequently, the reduction in levels of oleic acid and linoleic acid in fermented leaves leads to an overall improvement in sensory quality.

Free amino acids are important nitrogen compounds and key contributors to the aromatic compounds of cigar tobacco. However, little knowledge is available about the changes in free amino acid profiles during the fermentation of cigar tobacco leaves. In this study, a comprehensive analysis was conducted on 19 free amino acids (Fig. 2B, Additional file 1: Table S2). The total content of free amino acids shows a negative correlation with sensory quality (Guo et al. 2018). The total amino acids content significantly decreased from 14.53 ± 0.56 mg/g and 26.60 ± 0.38 mg/g in H1 and S1 to 8.74 ± 0.97 mg/g and 21.58 ± 1.92 mg/g in H5 and S5 (*p* < 0.05), representing 39.84% and 20.56% decreases, respectively, indicating an improvement in sensory quality. Maillard and condensation reactions are the probable reasons for the decline of amino acids in fermented leaves. For example, condensation reactions between amino acids and polyphenols or quinones result in the formation of water-insoluble complex products, which can act as catalysts for the oxidation of excess amino acids, leading to the decomposition of the amino acids into ammonia and α-keto acids (Frankenburg and Gottscho 1952; Hamilton and Lowe 1978). Notably, the content of amino acids varied in cigar tobacco leaves. Among them, Asp was the most abundant amino acid, accounting for more than 40% of the total amino acids content, which is different from flue-cured tobacco. Glu was identified as the second most abundant amino acid in cigar tobacco leaves, followed by Pro in Hainan tobacco leaves, which is consistent with previous studies (Ali et al. 2006; Kou et al. 2013). However, in Sichuan tobacco leaves, Pro was higher than Asp throughout the fermentation process. Tobacco variety may primarily account for this difference. This result shows the importance of characterizing the amino acid content of different varieties. Apart from Asp, Glu, and Pro, the contents of other amino acids in tobacco leaves were almost less than 1 mg/g. After fermentation, the levels of 11 amino acids decreased, Ala and Glu were included, these two amino acids have a negative effect on the sensory quality of tobacco leaves (Shen et al. 2022). In contrast, the levels of Leu and Iso significantly increased in both Hainan and Sichuan samples after 28 days of fermentation (*p* < 0.05). These changes likely improved the sensory quality of cigars, given that a high level of Leu has been reported to reduce offensive odor in tobacco leaves (Wang et al. 2002).

The sensory characteristics of tobacco leaves are additionally influenced by the composition and content of aromatic compounds. A total of 30 aromatic compounds were further analyzed based on GC–MS during the fermentation (Additional file 1: Table S3). The detected compounds were divided into 8 categories, including 15 ketones, 6 aldehydes, 4 alcohols, 1 pyrrole, 1 furan, 1 phenol, 1 eater, and 1 hydrocarbon, and these compounds exhibit a high frequency of detection and extensive academic research in tobacco leaves. The total amount of aromatic compounds initially increased in both Hainan and Sichuan tobacco leaves, reaching maximum values on the 14th day of fermentation with concentrations of 756.0 ± 18.53 μg/g and 720.55 ± 9.30 μg/g, respectively, then gradually decreased. These 30 volatile compounds can be classified into six groups based on their biosynthetic pathways, including the degradation of chlorophyll, carotenoid, phenylalanine, Siebel alkane, Maillard reaction products, and others (Fig. 2C). Among these compounds, neophytadiene, a degradation product of chlorophyll, emerged as the most abundant aromatic substance in cigars, accounting for over 60% of the total aromatic substances. Neophytadiene directly enters the flue gas, resulting in reduced smoke irritation and a smoother smoking experience. During the fermentation, the variation in neophytadiene displayed a similar trend to that observed for the overall quantity of aromatic components. The increase in neophytadiene content between 0 and 14 days mainly originated from phytol, a component generated by the degradation of chlorophyll (Hu et al. 2010). Conversely, the decline in neophytadiene content from 14th to 28th days was mainly due to the oxidation of neophytadiene, leading to its decomposition and conversion into low molecular weight compounds with a distinct alcohol flavor (Wahlberg et al. 1977). The next abundant group of compounds was carotenoid degradation products, which are less irritating and have a great impact on tobacco aroma. The contents of most carotenoid degradation products were less than 20 μg/g. It is known that the sensory effects of aromatic substances are not only affected by concentration and odor characteristics, but also closely related to odor threshold (Deng et al. 2021). Carotenoid degradation products have great effects on the whole flavors of tobacco leaves because of their low odor threshold (Wei et al. 2005), especially β-damascenone, which was identified as a key odorant in Pu-erh tea, fruits, and beverages (Deng et al. 2021). The content of β-damascenone significantly decreased after fermentation, and it showed an extremely low odor threshold in water according to a previous study (Liu et al. 2019), contributing more to the rosey and fruity aroma of cigars (Mookherjee and Wilson 1990; Pineau 2007). Isophorone, a compound known for its woody fragrance and previously identified as a modifying aromatic substance in cigar leaves (Yao et al. 2022), was nearly absent at the end of fermentation, indicating a decrease in the woody fragrance of cigar tobacco. The degradation products of phenylalanine, including benzyl alcohol, phenylethyl alcohol, benzaldehyde, and phenylacetaldehyde, showed floral odor of rose, strong almond, and honey-like odor (Wu et al. 2019). After fermentation, the total content of these four substances decreased by 17.79% and 38.72% in Hainan and Sichuan tobacco leaves, respectively. Benzaldehyde and phenylacetaldehyde can be converted into benzyl alcohol and phenylethyl alcohol, respectively. Furthermore, benzyl alcohol and phenylethyl alcohol can be further transformed into benzyl formate and 2-phenylethyl formate, which possess floral odors (Mookherjee and Wilson 1990). 5-Methyl furfural, a product of the Maillard reaction, contributes to sweetness and increases smoke concentration. The content of 5-methyl furfural increased by 286% and 70.4% after fermentation in Hainan and Sichuan tobacco leaves. During the fermentation of herbaceous Peony black tea, the content of 5-methyl furfural showed an upward trend after fermentation, and it imparts desirable sensory characteristics (Wang et al. 2023). It can be seen from the results that the contents of most aromatic compounds in tobacco leaves exhibited a decrease after the fermentation process. It is known that tobacco leaves encompass a wide range of aromatic components, and the degradation of aromatic substances may lead to the generation of novel aromatic compounds. Moreover, it is worth mentioning that unlike flue-cured tobacco, which predominantly contains neutral flavor substances, cigar tobacco is characterized by the prevalence of alkaline flavor substances. Therefore, further analysis is required to comprehensively examine the changes in alkaline aromatic components during cigar tobacco fermentation.

### Bacterial succession during the fermentation process of cigar tobacco leaves

**Fig. 3:**
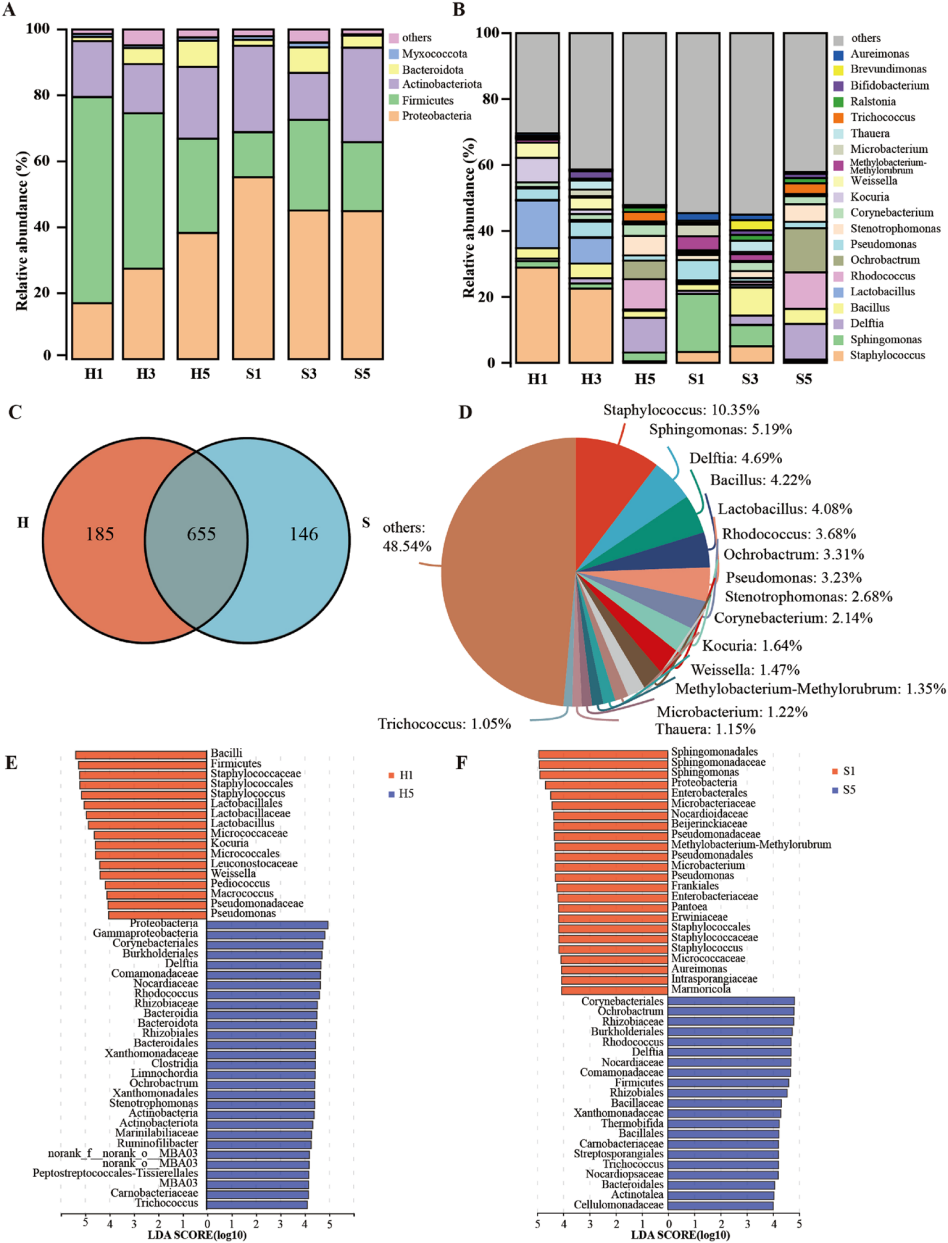
Analysis of bacterial community during the fermentation process. **A** Bar plot analysis of the bacterial community at the phylum level. **B** Bar plot analysis of the bacterial community at the genus level. **C** Venn diagram depicting shared and unique genera between Hainan and Sichuan samples. **D** Pieplot analysis of shared genera between Hainan and Sichuan samples. LEfSe analysis of Hainan **E** and Sichuan **F** bacterial communities that differed between pre- and post-fermentation stages based on LDA scores

DNA extraction and sequencing were performed on tobacco samples collected from Hainan and Sichuan at different fermentation stages, with a total of 947,395 sequences (ranging from 32,285 to 157,001) and an average sequence of 28,138 sequences per sample. The α-rarefaction curves indicated that the sequencing depth was sufficient for subsequent analysis (Additional file 1: Fig. S1). At the phylum level, Proteobacteria, Firmicutes, Actinobacteria, and Bacteroidetes were the dominant phyla in both regions (Fig. 3A), which is consistent with the findings of previous studies (Li et al. 2020; Liu et al. 2021b). However, the relative abundance of these phyla varied among regions and fermentation stages. In the fermentation of Hainan tobacco leaves, Firmicutes was the predominant phylum in the initial stage with a relative abundance of 62.56%, followed by Actinobacteria (16.98%), and Proteobacteria (16.95%). As fermentation progressed, Firmicutes decreased gradually, while Proteobacteria increased considerably, ultimately becoming the predominant phylum by the end (38.28%). Differently, Proteobacteria was consistently the dominant phylum throughout the fermentation process of Sichuan tobacco leaves, with relative abundances ranging from 44.90 to 55.22%, aligning with the findings of a previous study on the dynamic microbiota of Shiyan No.1 tobacco (collected from Sichuan, China) during fermentation (Li et al. 2020). Our study demonstrated great variations in phylum level bacterial among cigar tobacco leaves from different regions within the same country, consistent with previous research on cigar tobacco leaves from different countries (Zheng et al. 2022a).

At the genus level, a total of 840 and 801 genera were identified in the samples from Hainan and Sichuan, respectively. Venn diagrams were used to demonstrate the exclusive and shared genera between the two regions. There were 185 and 146 exclusive genera in Hainan and Sichuan cigar tobacco leaves, respectively, while 655 genera were common to both regions (Fig. 3C). The number of shared genera was far higher than the number of exclusive genera, indicating that there are notable similarities in the microbial community compositions in tobacco leaves between the two regions. Among the shared genera, *Staphylococcus* was the most abundant genus, followed by *Sphingomonas*, *Delftia*, *Bacillus*, *Lactobacillus*, *Rhodococcus*, *Ochrobactrum*, *Pseudomonas*, *Stenotrophomonas* and *Corynebacterium* (Fig. 3D). These shared genera were widely distributed throughout the fermentation, and their relative abundances were variable at different fermentation stages. In H1, *Staphylococcus* (28.94%), *Lactobacillus* (14.41%), *Kocuria* (7.36%), *Weissella* (4.56%), and *Pseudomonas* (3.53%) were the most dominant bacteria (Fig. 3B). In contrast, *Sphingomonas* (17.55%), *Pseudomonas* (6.18%)*, Methylobacterium–Methylorubrum* (4.26%), *Dyella* (3.99%), and *Microbacterium* (3.60%) were dominant in S1 (Fig. 3B). As fermentation proceeded, the relative abundance of bacteria in cigar tobacco leaves underwent significant changes. Notably, after fermentation, *Delftia* (10.59%, 10.76%)*, Rhodococcus* (9.15%, 11.00%), *Stenotrophomonas* (5.91%, 5.36%), and *Ochrobactrum* (5.60%, 13.44%) became dominant genera in both H5 and S5, suggesting they are more suitable for fermentation environment (Li et al. 2023).

LEfSe analysis was employed with a threshold LDA score of 4.0 to identify distinct bacteria taxa at different fermentation stages (Fig. 3E, F). The results showed that the dominant microorganisms mentioned above could potentially serve as biomarkers for pre- and post-fermentation stages, respectively. In addition to these dominant microorganisms, *Pediococcus* was identified as a biomarker for tobacco leaves before fermentation, which exhibited a decreasing trend along with the fermentation, and becoming undetectable by the end (Additional file 1: Fig. S2). *Pediococcus* has been detected in many fermentation materials (Zang et al. 2022), a similar changing trend during gray sufu fermentation was observed, and it showed a positive correlation with propionic acid (Ding et al. 2023). It is also known to be a functional genus that produces lactic acid during liquor fermentation (Du et al. 2019). *Ruminofilibacter* and *Actinotalea* were identified as biomarkers in H5 and S5, which were only presented in the H5 and S5 samples (Additional file 1: Fig. S2), with abundances of 3.87% and 0.16% in H5 and 1.01% and 2.28% in S5, respectively. *Ruminofilibacter* was more frequently detected in composting, and it was identified as the dominant predictor taxon for bacterial succession in composting (Xie et al. 2021). *Actinotalea*, belonging to the Cellulomonadaceae family, can produce bioactive compounds (Zhang et al. 2014). Although the functions of these genera in cigar tobacco leaves are still unclear, their succession trend may serve as a potential indicator of normal fermentation. In tobacco manufacturing, the fermentation process is typically controlled using empirical methods; changes in chemical substances and microbiota are often imperceptible, making it difficult to monitor the tobacco process by evaluating these changes (Wang et al. 2016). Furthermore, microbial markers could be employed to characterize the fermentation process and assist in developing a microbiota-based strategy for process monitoring (Tan et al. 2019).

### Dynamic changes of bacterial community structure during the fermentation process of cigar tobacco leaves

**Fig. 4:**
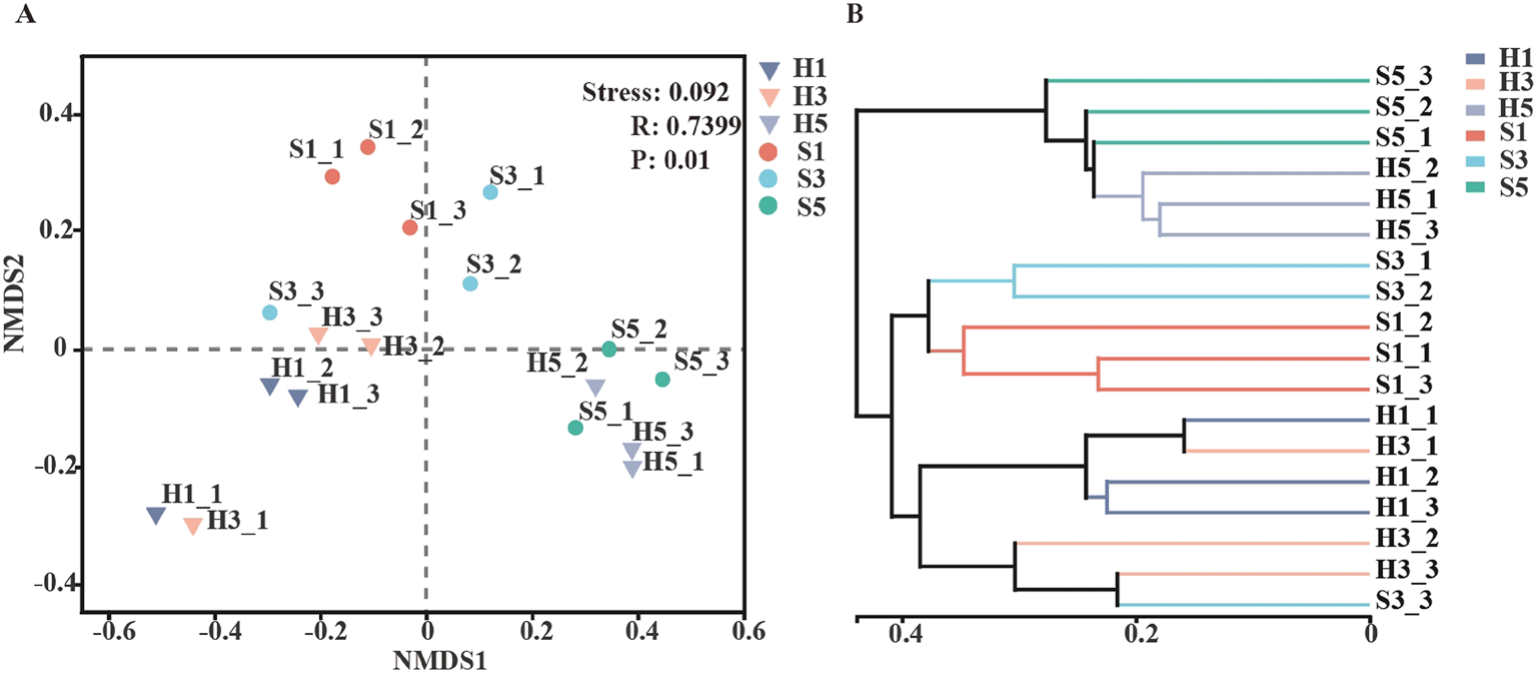
Analysis of the differences in bacterial succession during the fermentation process. **A** Non-metric multidimensional scaling analysis of samples at different fermentation stages. **B** Analysis of the distance between inter- and intra-groups at the genus level in different samples

To assess the influence of fermentation and geographical location on the bacterial community structure, cluster analysis and NMDS based on the Bray–curtis algorithm were conducted (Fig. 4A). The stress value was 0.092 (< 0.1), indicating that the NMDS analysis was reliable (Chen et al. 2019). Each point in the graph represents a sample replicate, and the distance between points indicates the degree of dissimilarity between them. The H1 and S1 points were distant from each other, revealing that bacterial community structure in the leaves before fermentation differed from each other. However, bacterial communities in the late stage of fermentation (H5 and S5) were similar to each other while being distant from other stages. The UPGMA cluster analysis revealed a clear separation of samples into two branches (Fig. 4B). H5 and S5 were clustered together in one branch, whereas H1, H3, S1, and S3 were clustered together in another branch. These results indicated that geographical origin had a great impact on the bacterial community structure (Salazar and Lizarazo-Medina 2021; Wagner et al. 2016), which is in line with previous research on tobacco-associated microbiota (Hu et al. 2022a; Xing et al. 2021). The fermentation process significantly altered the phyllosphere microbial community structure, and as fermentation progressed, geographical signatures became less apparent and the community structure underwent reshaping, eventually becoming similar after fermentation. Similar observations have been reported in studies on composting and food fermentation (Chen et al. 2019; Niccum et al. 2019; Zhao et al. 2019). High-quality cigars are characterized by a balanced chemical composition. The improvement in sensory properties of tobacco leaves after fermentation, meeting factory standards, is largely attributed to microbially induced chemical transformations. The composition and succession of phyllosphere microbial communities are crucial indicators for ensuring tobacco quality across different fermentation batches, which may explain the converging microbiota in tobacco leaves from different regions after fermentation.

### Correlations between bacterial community and flavor compounds

Spontaneous fermentation can hardly be controlled without understanding the relationship between microorganisms and flavor compounds in a dynamic perspective (Tan et al. 2019). Previous research has demonstrated the bacterial community, rather than the fungi community, primarily influences the flavor compounds of cigar tobacco leaves (Zheng et al. 2022a). However, limited knowledge exists about the specific impact of bacteria on flavor compounds during fermentation. In this study, we selected bacteria among the top 50 in relative abundance in Hainan and Sichuan tobacco leaves as the core microbiota, and they were set as the variable (x), while 59 flavor substances were set as an attributive variable (y). Then, the O2PLS model was used to analyze associations between the bacterial community and flavor compounds in samples from different regions. The Q2 values of the model were 0.587 and 0.788 for the Hainan and Sichuan samples, respectively, suggesting that the model was a good fit for analysis and prediction. To identify core functional bacteria in the fermentation process, three criteria were considered (Chen et al. 2020b; Li et al. 2019): (a) VIP value ≥ 1; (b) absolute value of correlation coefficient between the microbiota and flavors ≥ 0.7, *p* < 0.05; and (c) the bacteria were consistently detected throughout the fermentation process. Based on these criteria, six functional bacteria were identified in Hainan samples, including *Staphylococcus*, *Delftia*, *Rhodococcus*, *Ochrobactrum*, *Stenotrophomonas*, and *Kocuria* (Additional file 1: Fig. S3A). In the Sichuan samples, ten functional bacteria were identified, including *Ochrobactrum*, *Sphingomonas*, *Rhodococcus*, *Delftia*, *Pseudomonas*, *Stenotrophomonas*, *Methylobacterium–Methylorubrum*, *Bacillus*, *Dyella* and *Microbacterium* (Additional file 1: Fig. S3B). These microbes have previously been detected in tobacco leaves, but no studies have yet shown their function in fermented cigar tobacco leaves. In addition, these genera have been reported to play a significant role in the formation of flavor substances in fermented foods. For example, *Ochrobactrum* was one of the dominant bacteria in the spontaneous fermentation of Merlot wines (Liang et al. 2023). *Rhodococcus* was identified as a core bacterial genus in the fermentation of ‘Cabernet Sauvignon’ Wine in Ningxia, China (Zhang et al. 2022). *Delftia* and *Bacillus* were positively correlated with various flavor compounds in Nongxiangxing baijiu fermentation (Guan et al. 2023). *Kocuria* correlated with fermented Suanyu flavor formation (Zang et al. 2022). *Pseudomonas* contributed significantly to the diverse functions of Fuzhuan Brick tea (Li et al. 2019). *Stenotrophomonas* and *Rhodococcus* were significantly positively correlated with characteristic volatile flavor substances in fermented low-salt fish (Li et al. 2023), and *Staphylococcus* was associated with Cantonese soy sauce (Kuang et al. 2022). Although the presence of *Methylobacterium–Methylorubrum* in fermented food has not been reported, it has been reported as a dominant genus in cigar tobacco leaves (Jia et al. 2023). It is worth noting that functional bacteria in both regions include *Delftia*, *Rhodococcus*, *Ochrobactrum*, and *Stenotrophomonas*, and these four bacteria exhibited a strong correlation with amino acids, organic acids, and aromatic substances.

**Fig. 5:**
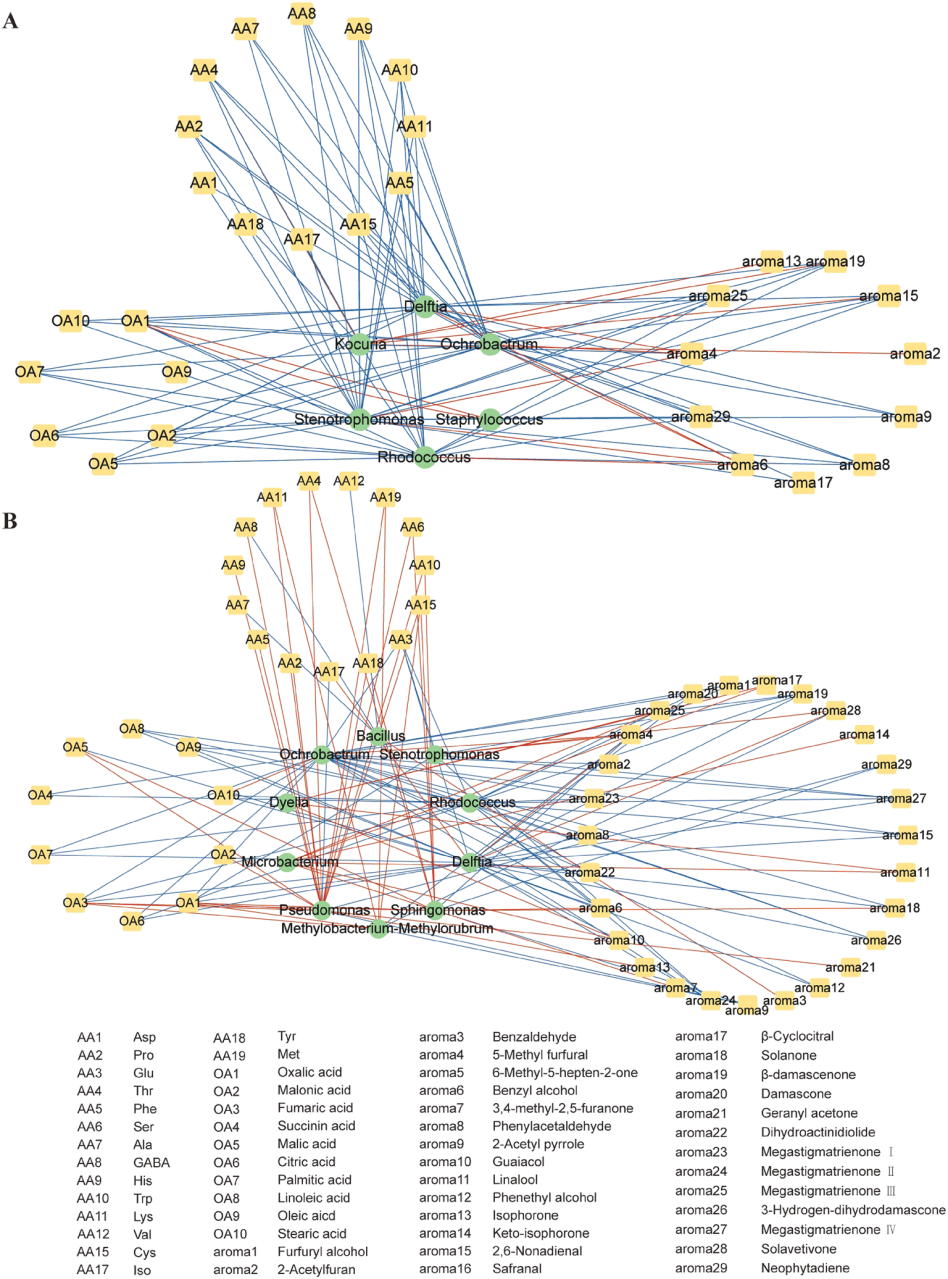
Correlation network of Hainan **A** and Sichuan **B** functional bacteria and flavor compounds during the fermentation. The green circles represent the functional bacteria, and the yellow squares represent the flavor compounds. The red lines indicate positive correlations, while the blue lines represent negative correlations

The correlation network between the functional bacteria and flavor substances was visualized using Cytoscape, revealing numerous relationships between bacteria and flavor substances (Fig. 5). *Stenotrophomonas* in Hainan leaves and *Delftia* in Sichuan leaves had the highest correlation coefficients with 25 and 24 flavor substances, respectively. Amino acids are easily degraded carbon sources and readily available to microorganisms (Zhu et al. 2016). *Stenotrophomonas* and *Delftia* showed a negative correlation with undesirable amino acids, such as Ala (in Hainan) and Glu (in Sichuan), indicating that these two genera promoted the degradation of amino acids and improved the quality of tobacco leaves. *Stenotrophomonas*, a common plant probiotic, has been widely studied for its ability to modulate amino acids content in fermented foods, such as Fu brick tea (Li et al. 2019), sweet tea (Huang et al. 2022), vinegar (Wang et al. 2016) and low-salt fish sauce (Li et al. 2023), etc. In addition, *Stenotrophomonas* has been reported to exhibit the ability to produce glutaminase, which effectively catalyzes the degradation of glutamine in soy sauce production (Wakayama et al. 2005). *Delftia* spp. are broadly distributed in the environment and have agricultural relevance (Bhat et al. 2022), the thriving growth of *Delftia* spp. on different amino acids provides strong evidence of their involvement in the transformation of amino acids within tobacco leaves (Juarez-Jimenez et al. 2010). Existing research on microorganisms of tobacco leaves paid little attention to *Stenotrophomonas* and *Delftia*, and the findings of this study are the first to recognize the important role they play in the amino acid metabolism of cigar tobacco leaves.

The correlation between bacteria and organic acids in tobacco leaves was mainly negative, emphasizing the significant role of bacteria in the metabolisms of organic acids during fermentation. Oxalic acid had the highest correlation coefficients with 5 bacteria in Hainan and 6 bacteria in Sichuan tobacco leaves, respectively. Among the correlations, *Delftia*, *Ochrobactrum*, *Stenotrophomonas*, and *Rhodococcus* showed negative correlation with oxalic acid, while *Sphingomonas*, *Pseudomonas, Methylobacterium–Methylorubrum*, and *Staphylococcus* exhibited a positive correlation with oxalic acid. Oleic acid and linoleic acid in tobacco leaves can increase irritation and reduce the smoothness of smoke (Hu et al. 2023). The negative correlation observed between *Delftia*, *Ochrobactrum*, and *Rhodococcus* with unsaturated fatty acids implies their potential role in improving sensory attributes. Furthermore, previous studies have reported that *Lactobacillus* is positively correlated with organic acid content during the food fermentation process (Zang et al. 2020). Our results indicated a positive correlation between *Lactobacillus* and oxalic acid, malic acid and citric acid, etc. (data not shown); however, the absolute value of the correlation coefficient was less than 0.6, suggesting that *Lactobacillus* may not be the main reason for the change of organic acid content in tobacco leaves.

A total of 11 aromatic substances were found to be associated with 6 bacteria in Hainan tobacco leaves, while 27 aromatic substances showed a correlation with 10 bacteria in Sichuan samples. *Ochrobactrum* displayed the highest correlation with 10 and 15 aromatic components in Hainan and Sichuan tobacco leaves, respectively, and it was positively correlated with 5-methyl furfural but negatively correlated with phenylacetaldehyde and 2, 6-nonadienal in both regions. In addition to *Ochrobactrum*, the results revealed a significant positive correlation between 5-methyl furfural and the genera *Delftia* and *Stenotrophomonas*. These microorganisms might be accountable for the elevation in 5-methyl furfural content during fermentation, suggesting their potential contribution to enhancing the sweetness of tobacco and enriching the smoke. Furthermore, *Bacillus* showed a positive correlation with the accumulation of benzaldehyde, indicating that it may contribute to the almond and cherry flavor of tobacco smoke. On one hand, *Bacillus* may facilitate the enzymatic conversion of phenylalanine to phenylpyruvic acid, which is further decarboxylated to generate benzaldehyde (Mathatheeranan et al. 2023); on the other hand, *Bacillus* has demonstrated its metabolic capacity to produce benzaldehyde, suggesting its involvement as a potential source of benzaldehyde (Koilybayeva et al. 2023). Moreover, *Microbacterium* displayed a positive correlation with 3,4-methyl-2,5-furanone, guaiacol, linalool, keto-isophorone, and β-cyclocitral in Sichuan tobacco leaves, these compounds exhibit desirable sensory characteristics, such as floral and fruity aroma, thereby suggesting that *Microbacterium* may contribute to the floral and fruity fragrance of tobacco. In Hainan tobacco leaves, *Kocuria* showed a positive correlation with 2-acetylfuran, isophorone, 2, 6-nonadienal, and β-damascenone. Similarly, *Kocuria* has been reported to be the functional microbe responsible for the production of amino acids and flavor compounds (i.e., phenylethanol, acetic acid) in many fermented foods, such as Suanyu, cheese, and soy sauce (Centeno et al. 2017; Tan et al. 2022; Zang et al. 2022). *Staphylococcus* displayed a low correlation coefficient with aromatic substances, potentially indicating that it is not directly involved in the synthesis and metabolism of aromatic substances. It has been found that *Staphylococcus* is involved in fat metabolism, and the resulting fatty acids are further degraded to aromatic substances (Zheng et al. 2022b). In addition, there are numerous negative correlations between microorganisms and aromatic constituents, and these aromatic compounds may be metabolized by microorganisms into new aromatic substances. 6-Methyl-5-heptene-2-one, safranal, and farnesyl acetone demonstrated a lower correlation with bacteria (|*r*|< 0.7), suggesting that the changes of these aromatic substances during fermentation may be attributed to natural physical changes. The interaction between microorganisms and flavor compounds greatly impacts the sensory quality of cigar tobacco leaves, resulting in the decrease of pungent and raw smell, along with the development of mild and aromatic smoke (Ning et al. 2023). Adding exogenous microorganisms as fermentation starters has proven to be an effective method in accelerating the fermentation process and improving the quality of fermented products, which has been widely applied in various products, such as tea, cheese, and Chinese liquor. Our results provide novel insights for mining functional microorganisms. Further studies should aim to isolate these functional microorganisms from tobacco leaves and confirm the accurate contribution of bacteria to enhance the overall quality of tobacco leaves.

### Bacterial co-occurrence network analysis

**Fig. 6:**
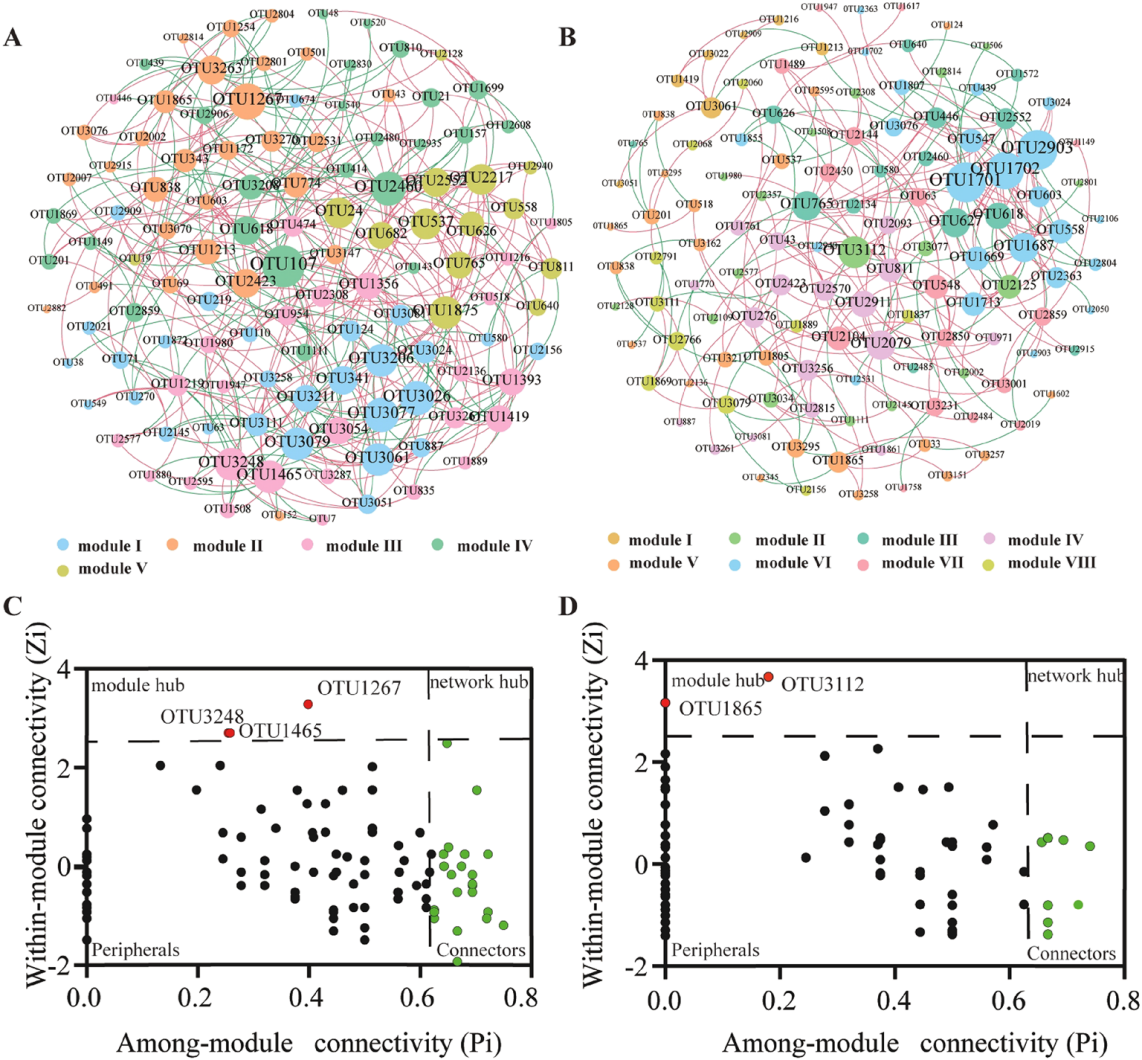
Co-occurrence network analysis for bacterial community during the fermentation process. The network of bacterial community for Hainan (**A**) and Sichuan (**B**) samples. Within or among module connectivity of each node for Hainan (**C**) and Sichuan (**D**) samples. The co-occurrence networks are colored by module. The size of each node is proportion to the number of connections (that is, degree). Red and green lines indicate positive and negative correlations, respectively

Microorganisms typically exist in complex communities rather than in isolation, and establish close interactions (Hughes et al. 2008). Microbial co-occurrence networks are extensively used to investigate relationships within microbial communities. By analyzing the topological properties of species within networks, keystone species can be identified (Proulx et al. 2005). In this study, a co-occurrence network was conducted to decipher potential interactions between microorganisms in the fermentation process. The bacterial network generated for Hainan and Sichuan tobacco bacterial communities consisted of 120 and 118 nodes (OTUs), respectively, with 420 (62.86% positive correlations) and 236 (70.89% positive correlation) edges (Fig. 6A, B). These networks demonstrated a prevalence of strong positive correlations among bacterial nodes, indicating that microorganisms cooperate to adapt to the phyllosphere environment and maintain the bacterial community structure (Jiang et al. 2022). The modularity index values for the Hainan and Sichuan networks were 0.468 and 0.639 (> 0.4), respectively, suggesting a typical modular structure with nonrandom patterns of microbial interactions in tobacco leaves (Newman 2006; Olesen et al. 2007). The nodes within the network were grouped into five modules for Hainan and eight major modules for Sichuan (Additional file 1: Figs. S4 and S5). *Bacillus*, belonging to the Bacillaceae family, was found in most modules. Based on the Zi and Pi values, the nodes were classified into peripherals (*Zi* ≤ 2.5, *Pi* ≤ 0.62), connectors (*Zi *≤ 0.25, *Pi* > 0.62), module hubs (*Zi* > 2.5, *Pi* ≤ 0.62), and network hubs (*Zi* > 2.5, *Pi* > 0.62) (Deng et al. 2012). Nodes categorized as connectors, module hubs, and network hubs within microbial co-occurrence networks are considered as keystone nodes that contribute to the sustainability and stability of the ecosystem, and are important for the bacterial community assembly and function, the removal of this keystone may have a significant impact on the community structure (Li et al. 2019; Steele et al. 2011). The majority of nodes in the ecological network were classified as peripherals in Hainan (75%) and Sichuan (88.98%) samples, respectively. A total of 27 (22.5%) and 11 (9.32%) nodes were identified as connectors. Three nodes (2.5%) were identified as module hubs in Hainan samples, including OTU1267 (*Sinibacillus*), OTU1465 (*Petrimonas*), and OTU3248 (*SM1A02*) (Fig. 6C). Two nodes (1.7%) were identified as module hubs in Sichuan samples, including OTU1865 (*Ureibacillus*) and OTU3112 (*Geobacillus*) (Fig. 6D). Network hubs were not identified in either sample. Among these module hubs, *Sinibacillus*, *Ureibacillus*, and *Geobacillus* belong to the family Bacillaceae, which is commonly found in tobacco leaves. Bacillaceae is known for its ability to degrade various compounds in tobacco leaves, such as protein, starch, and cellulose (Dai et al. 2020; Zhou et al. 2021), but its specific role in the co-occurrence network has not been elucidated. Our results revealed that this family may play an important role in the phyllosphere ecosystem. Additional file 1: Tables S4 and S5 display the relative abundances of connectors, with OTU 1980 (*Pseudomonas*) in Hainan at 1.42% and OTU 3211 (*Delftia*) in Sichuan at 4.8%, while the remaining connectors had relative abundances below 1%. Moreover, the relative abundances of all module hubs were less than 1%. Bacteria with relative abundances below 1% are identified as non-abundant bacteria (Peng et al. 2021). Thus, we infer that non-abundant species are essential for maintaining the ecological and functional stability within tobacco leaf microbial communities. It highlights the importance of considering non-abundant species alongside dominant ones when studying microbial communities in tobacco leaves. Similar observations have been reported in vinegar and Chinese strong-flavor liquor fermentation ecosystems (Peng et al. 2021; Zhao et al. 2022a).

## Conclusions

In summary, our results systematically characterized the conventional chemical composition, flavor substances, and bacterial succession during the fermentation of cigar tobacco leaves from different regions, and the associations between these factors were explored. The evidence leads to the conclusion that the structure and composition of bacterial community varied between sites before fermentation, and fermentation caused alterations. By the end of fermentation, cigar tobacco leaves from different regions exhibited a similar bacterial community structure. Moreover, 12 genera were identified as functional microorganisms that play crucial roles in amino acid, organic acid, and aromatic substance changes, and *Delftia*, *Rhodococcus*, *Ochrobactrum*, and *Stenotrophomonas* were included in both regions. Non-abundant species may play an important role in the maintenance of tobacco phyllosphere ecosystem sustainability and stability. These findings advance our understanding of the mechanisms underlying cigar tobacco fermentation and lay a solid foundation for enhancing the quality of cigar tobacco.

### Supplementary Information

**Additional file 1: Table S1.** Dynamic changes of organic acids during the fermentation process. **Table S2.** Dynamic changes of amino acids during the fermentation process. **Table S3.** Dynamic changes of aromatic compounds during the fermentation process. **Table S4.** Connectors in the bacterial co-occurrence network of Hainan tobacco leaves.**Table S5.** Connectors in the bacterial co-occurrence network of Sichuan tobacco leaves. **Figure S1.** Rarefaction curves of all samples. The abscissa represents the amount of randomly selected sequencing data; the ordinate represents the number of species observed. **Figure S2.** Core bacterial microbiome in Hainan and Sichuan samples and their distribution in different samples. **Figure S3.** VIP plot of the bacteria by O2PLS modeling during Hainan (**A**) and Sichuan (**B**) cigar tobacco fermentation. **Figure S4.** Hainan bacterial community composition of each co-occurrence network module. **Figure S5.** Sichuan bacterial community composition of each co-occurrence network module.

## Data Availability

Raw sequence reads can be obtained as fastq files from the Sequence Read Archive (SRA, https://www.ncbi.nlm.nih.gov/sra) under the BioProject accession number PRJNA880694.

## Abbreviations

O2PLS: Bidirectional orthogonal partial least squares
PCA: Principal component analysis
HCA: Hierarchical cluster analysis
PCR: Polymerase chain reaction
OTU: Operational taxonomic units
GC–MS: Gas chromatography–mass spectrometry
NMDS: Non-metric multidimensional scaling
UPG MA: Unweighted Pair-group Method with Arithmetic Mean
LEfSe: Linear discriminant analysis effect size
LDA: Linear discriminant analysis
Pi: Among-module connectivity
Zi: Within-module connectivity
Asp: Aspartic acid
Pro: Proline
Glu: Glutamic acid
Thr: Threonine
Phe: Phenylalanine
Ser: Serine
Ala: Alanine
GABA: γ-Aminobutyric acid
His: Histidine
Trp: Tryptophan
Lys: Lysine
Val: Valine
Arg: Arginine
Gly: Glycine
Cys: Cystine
Leu: Leucine
Iso: Isoleucine
Tyr: Tyrosine
Met: Methionine
