# Supplementary material for: The bacterial succession and its role in flavor compounds formation during the fermentation of cigar tobacco leaves
Source: Bioresour Bioprocess. 2023 Oct 31;10(1):74. doi: 10.1186/s40643-023-00694-9 (PMC10992852; doi:10.1186/s40643-023-00694-9)
Supplement: Supplementary file 1 — Additional file 1: Table S1. Dynamic changes of organic acids during the fermentation process. Table S2. Dynamic changes of amino acids during the fermentation process. Table S3. Dynamic changes of aromatic compounds during the fermentation process. Table S4. Connectors in the bacterial co-occurrence network of Hainan tobacco leaves.Table S5. Connectors in the bacterial co-occurrence network of Sichuan tobacco leaves. Figure S1. Rarefaction curves of all samples. The abscissa represents the amount of randomly selected sequencing data; the ordinate represents the number of species observed. Figure S2. Core bacterial microbiome in Hainan and Sichuan samples and their distribution in different samples. Figure S3. VIP plot of the bacteria by O2PLS modeling during Hainan (A) and Sichuan (B) cigar tobacco fermentation. Figure S4. Hainan bacterial community composition of each co-occurrence network module. Figure S5. Sichuan bacterial community composition of each co-occurrence network module. [file 40643_2023_694_MOESM1_ESM.docx]

**Additional material**

**The bacterial succession and its role in flavor compounds formation during the fermentation of cigar tobacco leaves**

Hongyang Si, Kun Zhou, Tingyi Zhao, Bing Cui*, Fang Liu, Mingqin Zhao*

*College of Tobacco Science, Flavors and Fragrance Engineering & Technology Research Center of Henan Province, Henan Agricultural University, Zhengzhou, Henan 450000, China*

*** Corresponding author**

Mingqin Zhao*****

[zhaomingqin@henau.edu.cn](mailto:zhaomingqin@henau.edu.cn)

Bing Cui*****

[bingcui@henau.edu.cn](mailto:bingcui@henau.edu.cn)

**Table S1** Dynamic changes of organic acids during the fermentation process.

**Table S2** Dynamic changes of amino acids during the fermentation process.

**Table S3** Dynamic changes of aromatic compounds during the fermentation process.

**Table S4** Connectors in the bacterial co-occurrence network of Hainan tobacco leaves.

**Table S5** Connectors in the bacterial co-occurrence network of Sichuan tobacco leaves.

**Figure** **S1** Rarefaction curves of all samples. The abscissa represents the amount of randomly selected sequencing data; the ordinate represents the number of species observed.

**Figure S2** Core bacterial microbiome in Hainan and Sichuan samples and their distribution in different samples.

**Figure S3** VIP plot of the bacteria by O2PLS modeling during Hainan (A) and Sichuan (B) cigar tobacco fermentation.

**Figure S4** Hainan bacterial community composition of each co-occurrence network module.

**Figure S5** Sichuan bacterial community composition of each co-occurrence network module.

**Table S1** Dynamic changes of organic acids during the fermentation process.

| **Organic acid (mg/g)** | **Hainan** | | | | | **Sichuan** | | | | |
| --- | --- | --- | --- | --- | --- | --- | --- | --- | --- | --- |
|  | **H1 (0d)** | **H2 (7d)** | **H3 (14d)** | **H4 (21d)** | **H5 (28d)** | **S1 (0d)** | **S2 (7d)** | **S3 (14d)** | **S4 (21d)** | **S5 (28d)** |
| Oxalic acid | 34.37±1.72b | 39.62±1.63a | 34.96±1.03b | 36.5±0.56b | 29.48±2.14c | 40.42±1.76a | 39.13±1.26a | 35.42±1.53b | 32.95±1.47b | 29.61±0.85c |
| Malic acid | 40.27±1.73c | 47.48±1.18a | 45.06±0.62b | 37.26±0.98d | 30.75±0.95e | 203.64±3.32a | 200.35±1.66a | 159.65±3.44c | 168.26±1.94b | 136.85±1.25d |
| Citric acid | 7.93±0.30c | 12.57±0.80a | 10.50±0.50b | 7.36±0.17c | 5.85±0.19d | 33.84±1.71a | 29.43±1.24bc | 28.52±0.55c | 30.73±0.28b | 17.24±0.98d |
| Malonic acid | 5.61±0.31a | 4.60±0.36b | 4.73±0.32b | 4.02±0.16c | 2.93±0.38d | 5.95±0.27a | 5.28±0.41b | 3.75±0.25c | 2.92±0.09d | 3.03±0.02d |
| Fumaric acid | 1.70±0.08b | 2.21±0.32a | 1.95±0.09ab | 2.11±0.19a | 1.65±0.10b | 6.27±0.24b | 6.82±0.15a | 4.96±0.08c | 4.89±0.09c | 3.69±0.21d |
| Succinic acid | 1.27±0.02d | 1.37±0.02c | 1.61±0.01a | 1.41±0.06c | 1.49±0.05b | 2.63±0.05a | 2.73±0.06ab | 2.56±0.03bc | 2.43±0.06c | 2.24±0.10d |
| Palmitic acid | 1.70±0.17a | 1.37±0.06b | 1.62±0.02a | 1.43±0.02b | 1.43±0.12b | 1.56±0.08bc | 1.88±0.03a | 1.41±0.09c | 1.58±0.14b | 1.48±0.30d |
| Linoleic acid | 0.77±0.02a | 0.61±0.03b | 0.74±0.05a | 0.66±0.02b | 0.73±0.04a | 0.53±0.02b | 0.64±0.06a | 0.59±0.06ab | 0.55±0.02b | 0.38±0.05c |
| Oleic acid | 3.55±0.08a | 2.70±0.16c | 3.44±0.10a | 2.99±0.20bc | 3.21±0.36ab | 2.68±0.21bc | 3.23±0.11a | 2.50±0.04c | 2.83±0.06b | 1.77±0.51d |
| Stearic acid | 0.50±0.05a | 0.42±0.02b | 0.49±0.02a | 0.45±0.01b | 0.43±0.01b | 0.50±0.04b | 0.58±0.06a | 0.42±0.04c | 0.48±0.01bc | 0.28±0.04d |
| Total | 97.67±0.97c | 112.95±2.28a | 105.1±0.81b | 94.19±1.19d | 77.9±2.3e | 298.02±3.86a | 290.07±2.42b | 239.78±4.76d | 247.62±2.81c | 196.57±1.69e |

Note: Values are mean ± standard error of the mean; (n=3). Statistical significance was assessed by one-way ANOVA followed by LSD multiple comparison test. Different lowercase letters indicate significant difference (p < 0.05).

**Table S2** Dynamic changes of amino acids during the fermentation process.

| **Amino**  **acid (mg/g)** | **Hainan** | | | | | **Sichuan** | | | | |
| --- | --- | --- | --- | --- | --- | --- | --- | --- | --- | --- |
|  | **H1 (0d)** | **H2 (7d)** | **H3 (14d)** | **H4 (21d)** | **H5 (28d)** | **S1 (0d)** | **S2 (7d)** | **S3 (14d)** | **S4 (21d)** | **S5 (28d)** |
| Asp | 7.07±0.18b | 12.62±1.57a | 11.18±0.27a | 6.71±0.55b | 4.82±0.61c | 10.67±1.1a | 9.82±0.99a | 10.15±1.13a | 10.04±0.92a | 11.11±1.12a |
| Pro | 1.18±0.01b | 0.81±0.07c | 1.27±0.06a | 0.80±0.03c | 0.56±0.11d | 3.52±0.38a | 3.02±0.45ab | 2.11±0.1c | 3.62±0.6a | 2.96±0.68bc |
| Glu | 1.33±0.08b | 1.54±0.01a | 1.36±0.05b | 1.21±013c | 1.15±0.05c | 2.75±0.02a | 2.37±0.41ab | 2.57±0.12a | 2.55±0.26a | 2.10±0.12b |
| Thr | 2.11±0.12a | 0.45±0.05b | 1.08±0.04c | 0.20±0.02d | 0.12±0.01c | 2.36±0.16a | 1.06±0.23b | 0.55±0.12c | 0.60±0.1c | 0.64±0.09c |
| Phe | 0.41±0.01b | 0.67±0.05a | 0.68±0.05a | 0.24±0.01c | 0.17±0.04d | 1.27±0.03a | 0.69±0.13b | 0.54±0.07bc | 0.47±0.14bc | 0.71±0.31c |
| Ser | 0.04±0.01d | 0.24±0.02b | 0.44±0.01a | 0.11±001c | 0.08±0.02c | 1.27±0.07a | 0.76±0.11b | 0.67±0.11b | 0.8±0.11b | 0.76±0.01b |
| Ala | 0.42±0.01b | 0.39±0.04b | 0.55±0.03a | 0.32±0.02c | 0.34±0.02c | 0.92±0.08a | 0.87±0.1a | 0.79±0.13a | 0.85±0.07a | 0.87±0.09a |
| GABA | 0.39±0.04ab | 0.34±0.02bc | 0.43±0.04a | 0.28±0.08cd | 0.24±0.03d | 0.84±0.02a | 0.78±0.22a | 0.62±0.1a | 0.76±0.06a | 0.66±0.06a |
| His | 0.39±0.01a | 0.36±0.03a | 0.35±0.05a | 0.12±0.02b | 0.08±0.01b | 0.55±0.05a | 0.25±0.12b | 0.19±0.08b | 0.17±0.07b | 0.15±005b |
| Trp | 0.26±0.02b | 0.30±0.02a | 0.22±0.02c | 0.06±002d | 0.03±0d | 0.49±0.03a | 0.19±0.01b | 0.10±0.01c | 0.04±0d | 0.03±0d |
| Lys | 0.20±0.02b | 0.32±0.04a | 0.34±0.01a | 0.16±0.03b | 0.11±0.01c | 0.45±0.15a | 0.36±0.19a | 0.38±0.02a | 0.40±0.08a | 0.40±0.02a |
| Val | 0.09±0.01c | 0.19±0.02a | 0.21±0.01a | 0.13±0.01b | 0.12±0.01b | 0.41±0.01a | 0.31±0.01c | 0.33±0.05c | 0.38±0.02ab | 0.35±0bc |
| Arg | 0.11±0.01b | 0.18±0a | 0.20±0.02a | 0.12±0.02b | 0.10±0.02b | 0.23±0.03a | 0.20±0.02a | 0.24±0.04a | 0.23±0.03a | 0.22±0.02a |
| Gly | 0.13±0.03a | 0.35±0.3a | 0.22±0.05a | 0.18±0.03a | 0.18±0.02a | 0.22±0.04a | 0.25±0.01a | 0.25±0.06a | 0.25±0.05a | 0.26±0.06a |
| Cys | 0.17±0.01ab | 0.19±0.02a | 0.17±0.02ab | 0.15±0.01b | 0.12±0.02c | 0.20±0.02a | 0.16±0.04ab | 0.14±0.02b | 0.15±0.01b | 0.14±0.02b |
| Leu | 0.06±0a | 0.18±0.01b | 0.20±0.01a | 0.11±0c | 0.11±0.01c | 0.17±0.01c | 0.19±0.01bc | 0.23±0.03a | 0.22±0.02ab | 0.20±0.01ab |
| Iso | 0.02±0b | 0.08±0.01a | 0.10±0.01a | 0.08±0.01a | 0.09±0.03a | 0.10±0.01c | 0.11±0c | 0.13±0b | 0.38±0.01a | 0.14±0.01b |
| Tyr | 0.06±0.02d | 0.12±0.01c | 0.20±0.02b | 0.11±0.01c | 0.24±0.02a | 0.10±0a | 0.06±0.01c | 0.07±0.01c | 0.8±0.01b | 0.06±0.01c |
| Met | 0.10±0.01a | 0.06±0.01b | 0.08±0.01ab | 0.06±0.01b | 0.07±0.02b | 0.09±0.02a | 0.06±0.03a | 0.06±0.02a | 0.09±0.02a | 0.06±0a |
| Total | 14.53±0.56b | 19.18±1.56a | 19.88±0.47a | 11.15±0.98c | 8.74±0.97d | 26.60±0.38a | 21.51±0.71b | 20.11±0.49b | 22.19±2.27b | 21.58±1.92b |

Note: Values are mean ± standard error of the mean; (n=3). Statistical significance was assessed by one-way ANOVA followed by LSD multiple comparison test. Different lowercase letters indicate significant difference (p < 0.05).

**Table S3** Dynamic changes of aromatic compounds during the fermentation process.

| **Compounds (μg/g)** | | **Hainan** | | | **Sichuan** | | |
| --- | --- | --- | --- | --- | --- | --- | --- |
|  |  | **H1 (0 d)** | **H3 (14 d)** | **H5 (28 d)** | **S1 (0 d)** | **S3 (14 d)** | **S5 (28 d)** |
| Degradation products of chlorophyll | Neophytadiene | 431.19±16.52b | 505.28±12.80a | 346.25±11.14c | 472.81±3.41c | 543.67±7.26a | 506.61±4.05b |
| Degradation products of carotenoid | 6-Methyl-5-hepten-2-one | 1.80±0.44a | 1.20±0.07a | 1.68±0.19a | 2.75±0.26a | 2.75±0.13a | 2.23±0.25a |
|  | Isophorone | 1.49±0.11a | 0b | 0b | 0.93±0.06a | 0.15±0.001b | 0c |
|  | Keto-isophorone | 0.40±0.02a | 0.44±0.04a | 0.40±0.03a | 0.50±0.06a | 0.30±0.03b | 0.22±0.01b |
|  | β-Cyclocitral | 0.47±0.02ab | 0.54±0.06a | 0.36±0.03b | 0.23±0.04a | 0.20±0a | 0.19±0.01a |
|  | β-Damascenone | 22.20±0.51a | 17.02±0.44b | 13.84±0.32c | 17.17±0.26a | 13.15±1.16b | 4.07±0.17c |
|  | β-Dihydrodamarone | 12.33±0.26b | 13.87±0.31a | 11.94±0.14b | 10.70±0.31a | 8.44±0.32b | 5.29±0.23c |
|  | Geranyl acetone | 3.74±0.37a | 3.61±0.29a | 3.65±0.1a | 4.09±0.18a | 2.40±0.12b | 2.67±0.34b |
|  | Dihydroactinidiolide | 5.41±0.25a | 6.17±0.3a | 6.38±0.33a | 10.73±0.45a | 10.20±0.15a | 6.15±0.35b |
|  | Megastigmatrienone I | 5.27±0.24b | 7.07±0.40a | 5.21±0.14b | 4.44±0.25a | 3.53±0.29a | 1.64±0.53b |
|  | Megastigmatrienone Ⅱ | 21.83±0.41b | 26.29±0.06a | 22.54±0.71b | 17.80±0.04a | 14.15±0.33b | 5.50±0.22c |
|  | Megastigmatrienone Ⅲ | 4.06±0.13b | 5.48±0.29a | 2.73±0.26c | 1.34±0.20a | 1.10±0.1ab | 0.80±0.09b |
|  | Megastigmatrienone Ⅳ | 25.22±2.11b | 30.67±3.07a | 25.60±0.88b | 5.29±0.33a | 4.92±0.09a | 1.87±0.34b |
|  | 3-Hydrogen-dihydrodamascone | 5.13±0.11a | 6.32±0.5a | 5.11±0.29a | 18.18±0.26a | 14.10±0.58b | 6.72±0.30c |
|  | Farnesyl acetone | 9.77±0.36b | 14.62±0.34a | 11.6±0.72b | 11.36±0.31a | 12.27±0.43a | 11.38±0.40 |
| Degradation products of Phenylalanine | Benzyl alcohol | 7.81±0.38b | 7.45±0.41b | 9.86±0.74a | 16.25±0.32a | 12.00±0.58b | 6.59±0.26c |
|  | Phenethyl alcohol | 14.22±0.62b | 15.36±0.34a | 14.44±1.22b | 15.52±0.23a | 12.63±0.36b | 9.09±0.38c |
|  | Benzaldehyde | 5.07±0.37a | 4.95±0.14a | 4.41±0.27a | 3.56±0.23b | 5.81±0.47a | 3.95±0.31b |
|  | Phenylacetaldehyde | 17.07±1.87b | 19.57±1.16a | 7.60±0.43c | 11.24±0.33a | 12.33±0.28a | 8.92±0.46b |
| Degradation products of Siebel alkane | Solanone | 38.30±2.9b | 53.73±3.81a | 34.10±0.4c | 37.99±2.74a | 30.90±0.59b | 23.58±0.08c |
| Maillard reaction products | Furfuryl alcohol | 2.70±0.27a | 2.56±0.27a | 3.46±0.41a | 5.57±0.12a | 4.63±0.36a | 3.01±0.4b |
|  | 2-Acetylfuran | 0.74±0.06a | 0.17±0.02b | 0.18±0.01b | 0.37±0.04a | 0.23±0.03b | 0.12±0.01c |
|  | 5-Methyl furfural | 3.43±0.3c | 8.89±0.30b | 12.50±0.24a | 2.50±0.26c | 3.11±0.07b | 4.26±0.12a |
|  | 3,4-methyl-2,5-furanone | 1.56±0.11a | 1.33±0.24a | 1.82±0.16a | 3.35±0.29a | 3.0±0.06ab | 2.50±0.05b |
|  | 2-Acetyl pyrrole | 0.13±0.03a | 0.13±0.02a | 0.07±0b | 0.13±0.03a | 0.18±0.02a | 0.18±0a |
| Others | Linalool | 1.04±0.44a | 1.19±0.17a | 0.89±0.36a | 2.46±0.47a | 1.89±0.19ab | 1.24±0.04b |
|  | 2, 6-Nonadienal | 0.35±0.02a | 0.30±0.01b | 0.21±0.01c | 1.07±0.08a | 0.89±0.05b | 0.53±0.05c |
|  | Guaiacol | 0.79±0.31a | 0.93±0.19a | 0.82±0.06a | 1.25±0.26a | 0.97±0.10ab | 0.63±0.02b |
|  | Solavetivone | 0.52±0.06b | 0.73±0.02a | 0.51±0.01b | 0.37±0.06a | 0.49±0.01ab | 0.65±0.08a |
|  | Safranal | 0.1±0.01a | 0.13±0.02a | 0.08±0.03a | 0.18±0.03a | 0.16±0.01a | 0.15±0.03a |
| Total |  | 644.14±6.11b | 756.00±18.53a | 548.25±4.79c | 680.13±6.22b | 720.55±9.30a | 620.74±7.00c |

Note: Values are mean ± standard error of the mean; (n=3). Statistical significance was assessed by one-way ANOVA followed by LSD multiple comparison test. Different lowercase letters indicate significant difference (p < 0.05).

**Table S4** Connectors in the bacterial co-occurrence network of Hainan tobacco leaves.

| **OTU** | **No. module** | **Zi** | **Pi** | **Genus** | **Relative abundance (%)** |
| --- | --- | --- | --- | --- | --- |
| OTU19 | 2 | -1.92961 | 0.666667 | Comamonas | 0.16 |
| OTU1980 | 3 | -0.52375 | 0.666667 | Pseudomonas | 1.42 |
| OTU2308 | 3 | -0.1654 | 0.65625 | Bacillus | 0.85 |
| OTU2595 | 3 | -0.88211 | 0.625 | Romboutsia | 0.75 |
| OTU603 | 4 | -1.05328 | 0.72 | Microbacterium | 0.74 |
| OTU518 | 3 | -0.88211 | 0.625 | Cutibacterium | 0.68 |
| OTU3261 | 3 | -0.1654 | 0.65625 | Corynebacterium | 0.41 |
| OTU2460 | 1 | 1.542816 | 0.702222 | Massilia | 0.32 |
| OTU674 | 0 | -1.19452 | 0.75 | Bacteroides | 0.24 |
| OTU2935 | 1 | -1.30546 | 0.666667 | Labilithrix | 0.23 |
| OTU1875 | 2 | 0 | 0.642857 | Thauera | 0.23 |
| OTU270 | 0 | -1.19452 | 0.75 | Enterococcus | 0.21 |
| OTU43 | 4 | -1.05328 | 0.625 | Cloacibacterium | 0.19 |
| OTU1356 | 3 | -0.1654 | 0.694215 | unclassified_f__Rhizobiaceae | 0.18 |
| OTU954 | 3 | -0.52375 | 0.693878 | Enterobacter | 0.17 |
| OTU107 | 1 | 2.492241 | 0.648199 | unclassified_f__Rhizobiaceae | 0.17 |
| OTU343 | 4 | 0.247831 | 0.641975 | Tumebacillus | 0.16 |
| OTU2423 | 4 | 0.247831 | 0.722222 | Lawsonella | 0.16 |
| OTU537 | 2 | 0.385922 | 0.650888 | Parafrigoribacterium | 0.16 |
| OTU580 | 0 | -0.92479 | 0.625 | Kocuria | 0.16 |
| OTU682 | 2 | -0.38592 | 0.694215 | Balneola | 0.14 |
| OTU2859 | 1 | -0.35603 | 0.693878 | Burkholderia-Caballeronia-Paraburkholderia | 0.13 |
| OTU2128 | 2 | -1.92961 | 0.666667 | Clostridium_sensu_stricto_1 | 0.13 |
| OTU474 | 3 | -0.1654 | 0.65625 | Tumebacillus | 0.13 |
| OTU24 | 2 | 0 | 0.674556 | norank_f__Rhizobiaceae | 0.12 |
| OTU71 | 0 | -0.92479 | 0.722222 | Solirubrobacter | 0.12 |
| OTU838 | 4 | 0.247831 | 0.68 | Arthrobacter | 0.11 |

**Table S5** Connectors in the bacterial co-occurrence network of Sichuan tobacco leaves.

| **OTU** | **No.Module** | **Zi** | **Pi** | **Genus** | **Relative abundance (%)** |
| --- | --- | --- | --- | --- | --- |
| OTU3211 | 0 | -0.14704 | 0.625 | Delftia | 4.80 |
| OTU1855 | 5 | -1.14485 | 0.666667 | Klenkia | 0.76 |
| OTU2815 | 3 | -0.79077 | 0.625 | Pseudokineococcus | 0.57 |
| OTU626 | 2 | -0.79862 | 0.72 | Nitrosomonas | 0.42 |
| OTU1805 | 0 | -0.14704 | 0.625 | Massilia | 0.37 |
| OTU2125 | 1 | 0.470592 | 0.693878 | Alicyclobacillus | 0.19 |
| OTU1572 | 2 | -1.3754 | 0.666667 | Aureimonas | 0.14 |
| OTU2911 | 3 | 0.431331 | 0.65625 | norank_f__67-14 | 0.13 |
| OTU3162 | 0 | -0.80874 | 0.666667 | unclassified_c__  Gammaproteobacteria | 0.13 |
| OTU3295 | 0 | 0.51465 | 0.666667 | Georgenia | 0.12 |
| OTU765 | 2 | 0.354943 | 0.74 | norank_f__Steroidobacteraceae | 0.12 |


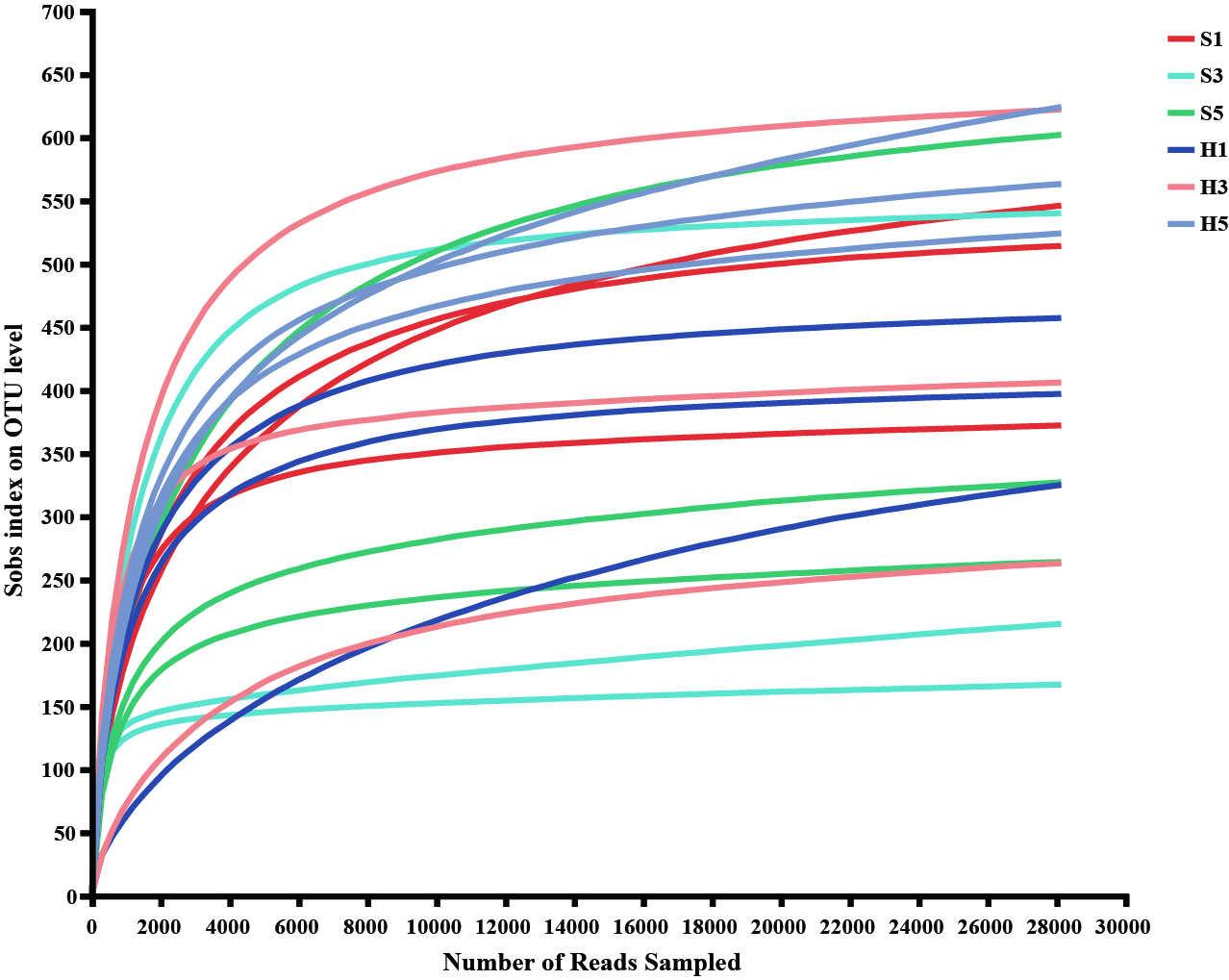

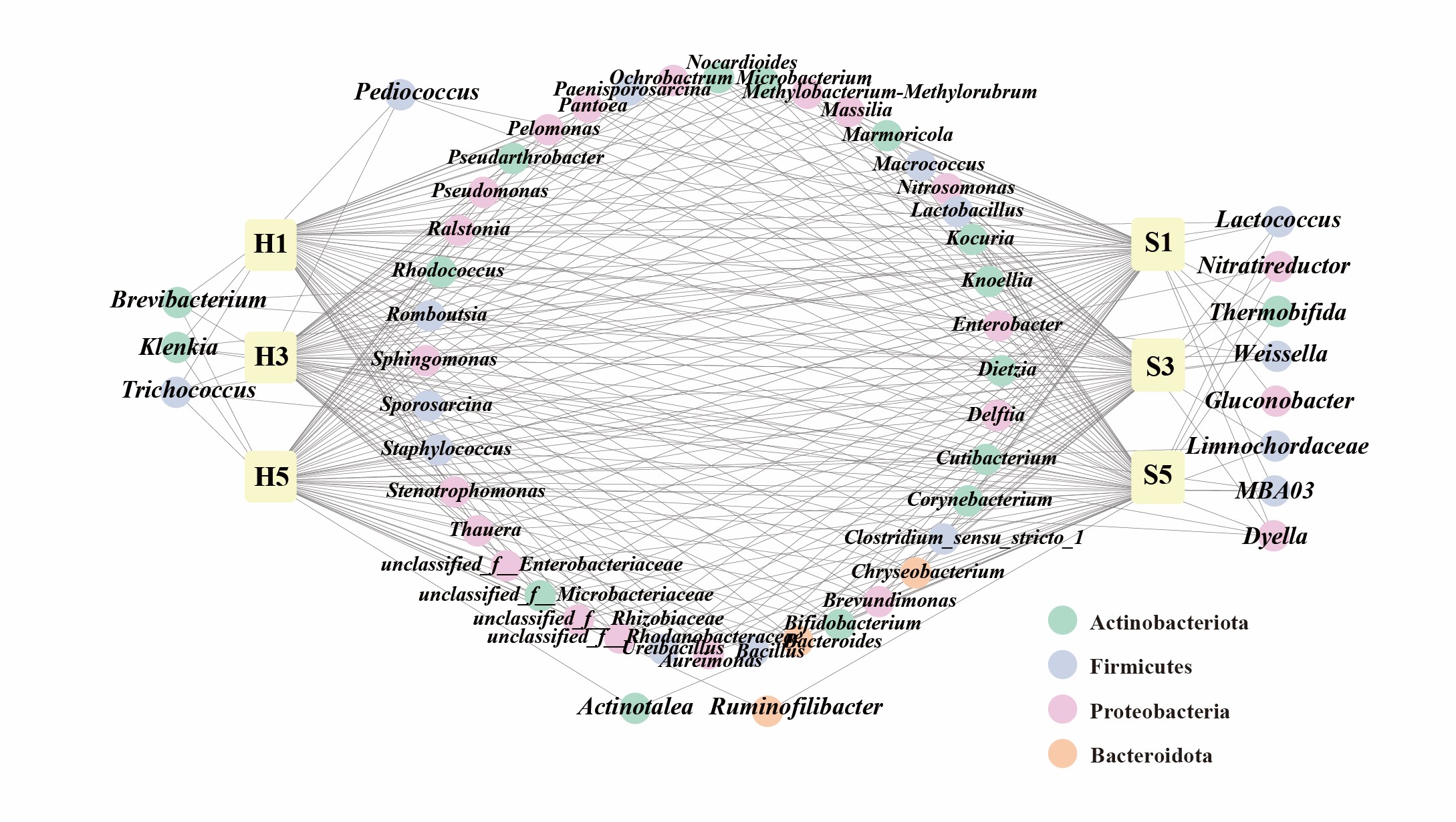


**Figure** **S1** Rarefaction curves of all samples. The abscissa represents the amount of randomly selected sequencing data; the ordinate represents the number of species observed.

**Figure S2** Core bacterial microbiome in Hainan and Sichuan samples and their distribution in different samples.

**
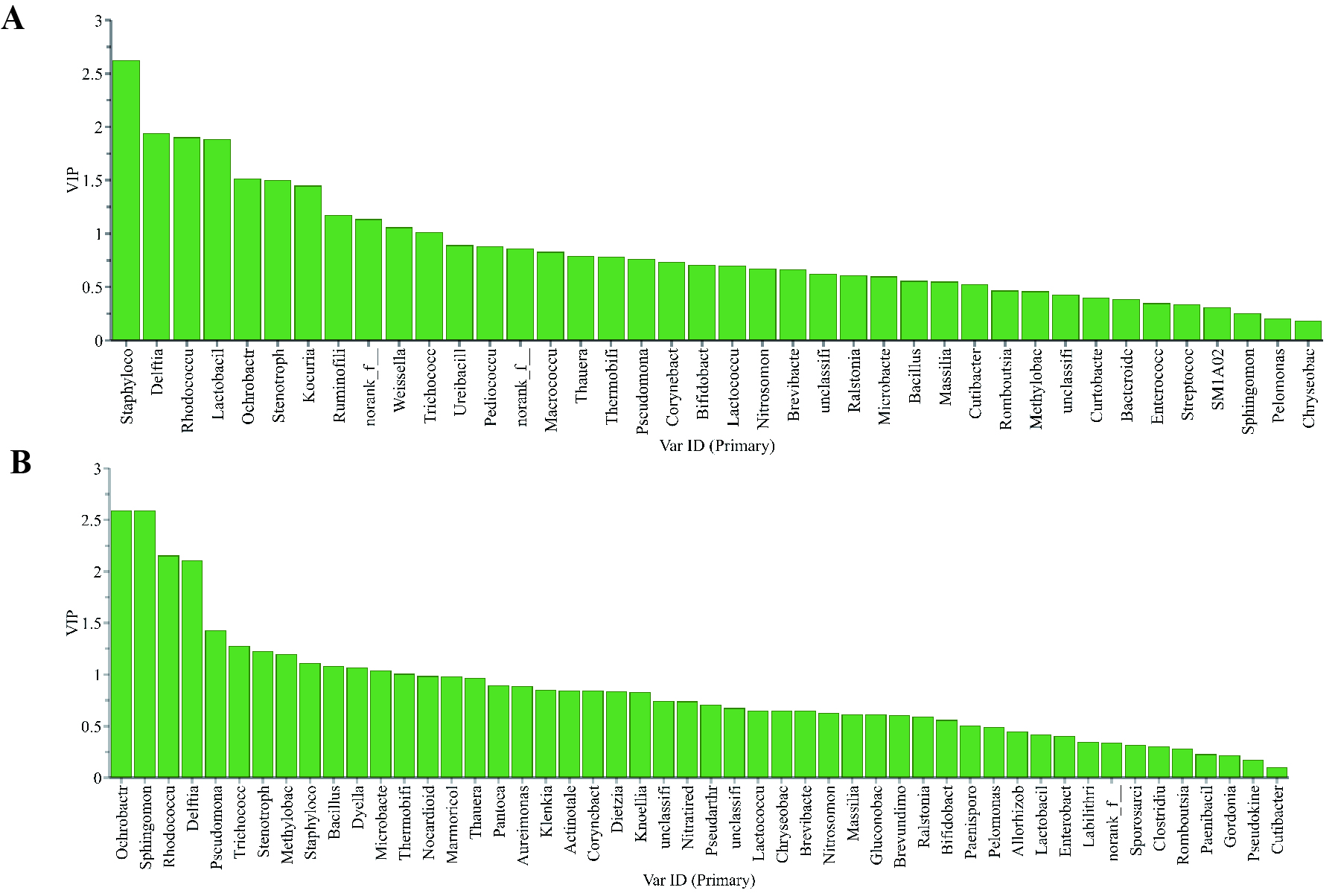
**

**Figure S3** VIP plot of the bacteria by O2PLS modeling during Hainan (A) and Sichuan (B) cigar tobacco fermentation.


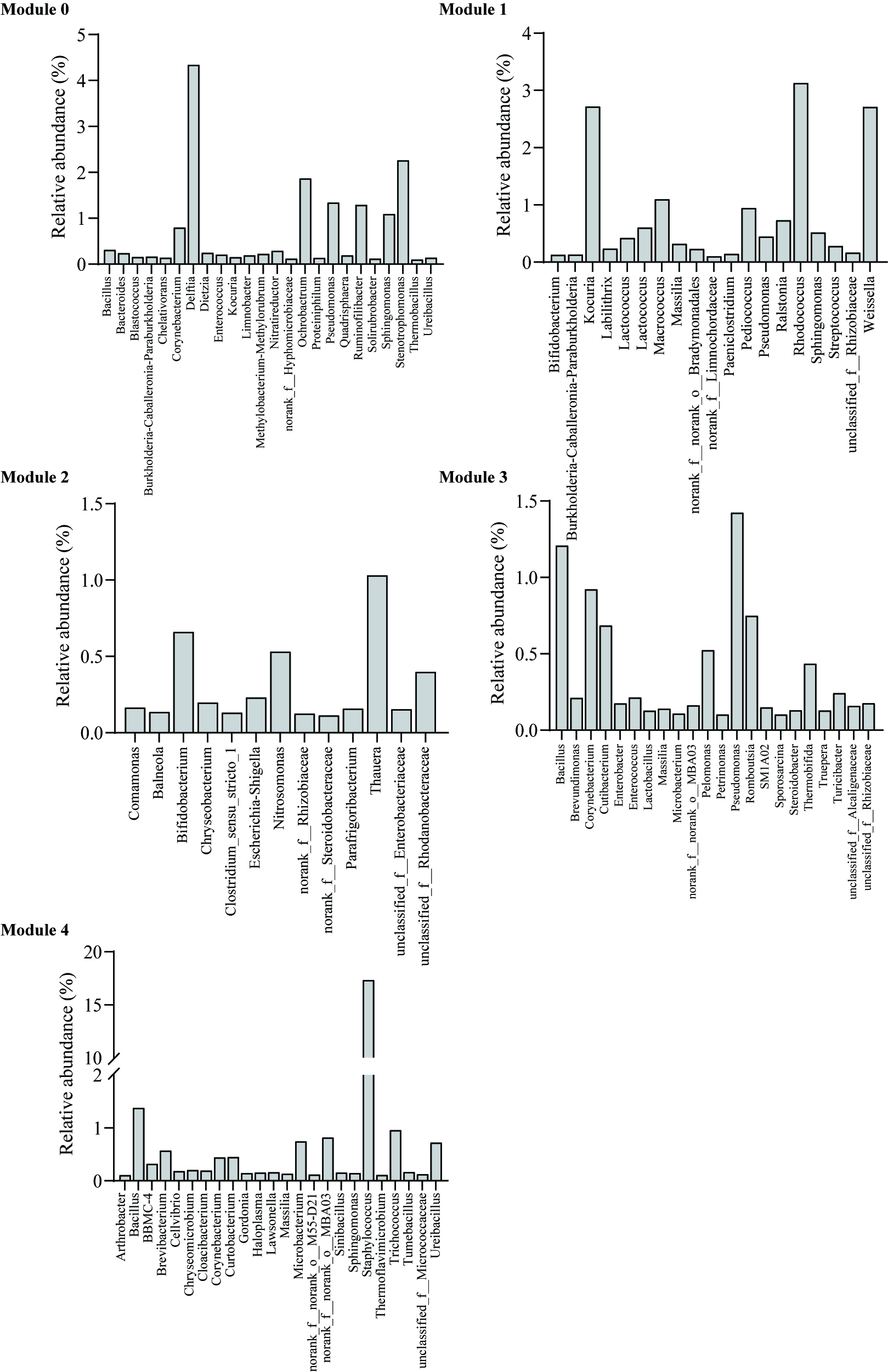


**Figure S4** Hainan bacterial community composition of each co-occurrence network module.

**
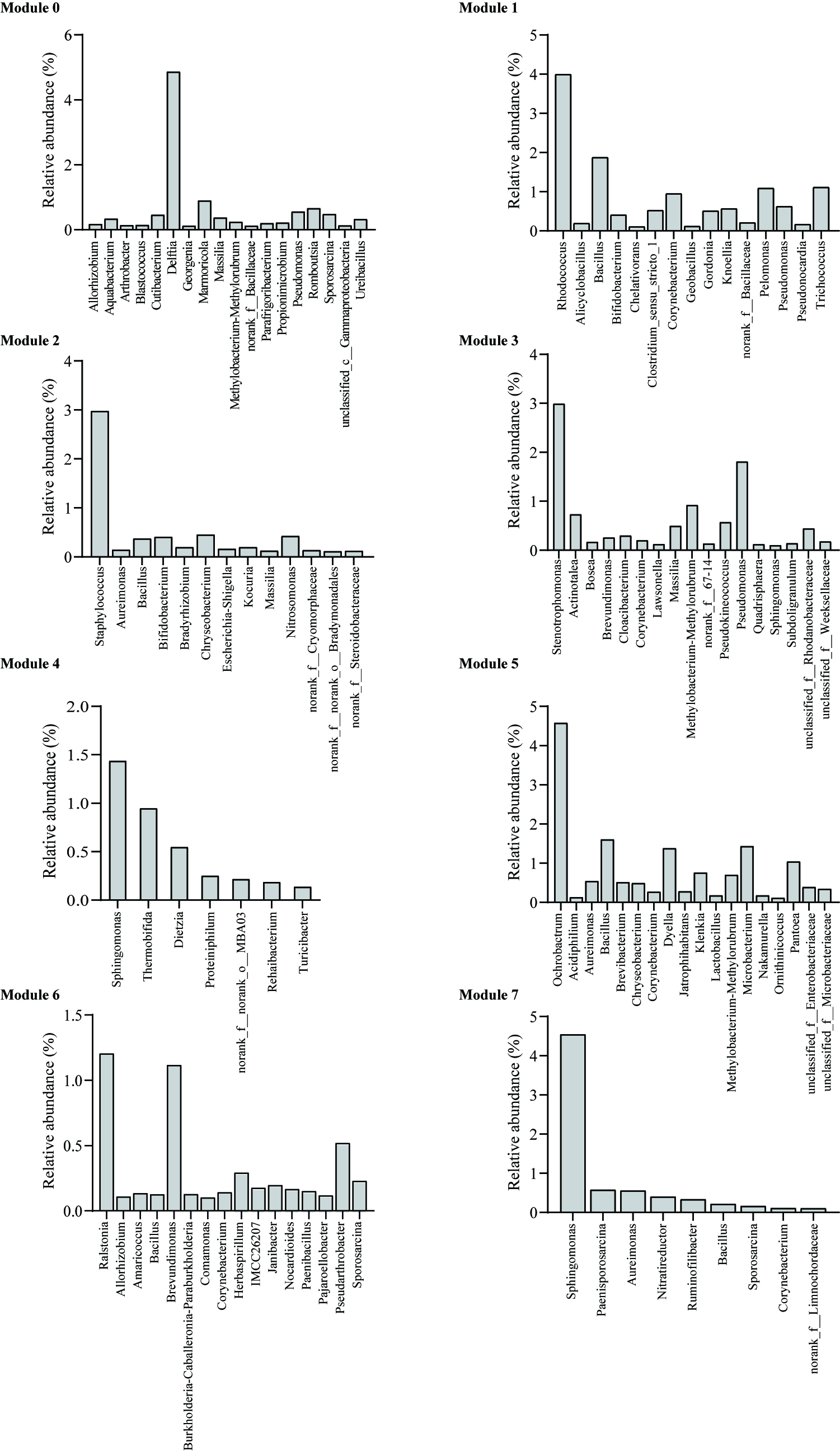
**

**Figure S5** Sichuan bacterial community composition of each co-occurrence network module.
